# The SOCS-Box of HIV-1 Vif Interacts with ElonginBC by Induced-Folding to Recruit Its Cul5-Containing Ubiquitin Ligase Complex

**DOI:** 10.1371/journal.ppat.1000925

**Published:** 2010-06-03

**Authors:** Julien R. C. Bergeron, Hendrik Huthoff, Dennis A. Veselkov, Rebecca L. Beavil, Peter J. Simpson, Stephen J. Matthews, Michael H. Malim, Mark R. Sanderson

**Affiliations:** 1 Department of Infectious Diseases, King's College London School of Medicine, London, United Kingdom; 2 Randall Division of Cell and Molecular Biophysics, King's College London, London, United Kingdom; 3 Division of Molecular Biosciences, Imperial College London, London, United Kingdom; Duke University Medical Center, United States of America

## Abstract

The HIV-1 viral infectivity factor (Vif) protein recruits an E3 ubiquitin ligase complex, comprising the cellular proteins elongin B and C (EloBC), cullin 5 (Cul5) and RING-box 2 (Rbx2), to the anti-viral proteins APOBEC3G (A3G) and APOBEC3F (A3F) and induces their polyubiquitination and proteasomal degradation. In this study, we used purified proteins and direct *in vitro* binding assays, isothermal titration calorimetry and NMR spectroscopy to describe the molecular mechanism for assembly of the Vif-EloBC ternary complex. We demonstrate that Vif binds to EloBC in two locations, and that both interactions induce structural changes in the SOCS box of Vif as well as EloBC. In particular, in addition to the previously established binding of Vif's BC box to EloC, we report a novel interaction between the conserved Pro-Pro-Leu-Pro motif of Vif and the C-terminal domain of EloB. Using cell-based assays, we further show that this interaction is necessary for the formation of a functional ligase complex, thus establishing a role of this motif. We conclude that HIV-1 Vif engages EloBC via an induced-folding mechanism that does not require additional co-factors, and speculate that these features distinguish Vif from other EloBC specificity factors such as cellular SOCS proteins, and may enhance the prospects of obtaining therapeutic inhibitors of Vif function.

## Author Summary

HIV-1 is the etiologic agent of AIDS. Current therapies are based on cocktails of anti-viral drugs that inhibit viral enzymes essential for virus replication, but this strategy has several shortcomings, including the development of drug-resistant virus strains. Consequently, pharmacologic strategies that interfere with additional aspects of HIV-1 replication have the potential to enhance HIV-1 treatments. The HIV-1 Vif protein is a promising target for the development of new anti-HIV-1 therapeutics; it functions to counteract the cellular proteins A3G and A3F, two components of a human anti-viral defence mechanism. Vif accomplishes this by hijacking a cellular complex (comprising the proteins EloB, EloC, Cul5 and Rbx2), which then eliminates A3G and A3F from infected cells by degradation, therefore evading their anti-viral effect. Here, we used purified proteins to reconstitute *in vitro* the recruitment of this complex by HIV-1 Vif. Using structural and biochemical methods, we dissected the different events involved in Vif's interaction with the EloBC complex. Our results reveal fundamental differences with cellular proteins known to recruit this complex, suggesting that Vif possesses unique features that could be targeted by pharmacologic intervention, without disturbing normal cell functions. The assays reported here could be utilized for the discovery of such inhibitors.

## Introduction

The cullin-RING ubiquitin ligases form one of the largest family of E3 ubiquitin ligases, and members are implicated in many cellular functions such as the cell cycle, signal transduction, transcription, circadian clock, development, DNA replication and protein quality control [1]. All members of this family are composed of a cullin E3 core and a specificity factor, which will select the appropriate substrate for ubiquitylation [2]. One such E3 core is composed of elongin B (EloB), elongin C (EloC), cullin 5 (Cul5) and RING-box protein 2 (Rbx2). A large number of specificity factors can associate with this core [3] via a so-called SOCS-box, composed of a BC-box responsible for binding to EloBC, followed by a cullin-box responsible for selecting Cul5 [4]. The crystal structures of the specificity factors SOCS2 [5] and SOCS4 [6] in complex with the EloB-EloC heterodimer (EloBC) have shown that the BC-box forms an α-helix, which binds to EloC via specific hydrophobic interactions. The consensus cullin-box sequence is composed of a Leu-Pro-Leu-Pro (LPLP) motif followed by a α-helix presenting a hydrophobic face, but the mechanism by which it specifies Cul5 engagement remains to be elucidated. Importantly, no system has yet been reported to study the *in vitro* binding of specificity factors to EloBC. Indeed, it has been shown that one such specificity factor, SOCS3, does not bind to EloBC when purified separately, but requires co-expression to form a SOCS3-EloBC ternary complex [7].

**Figure 1:**
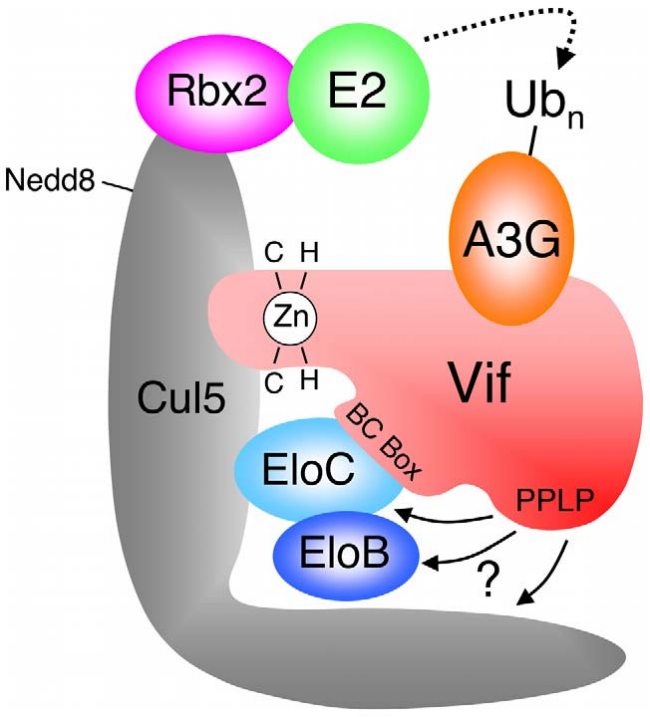
HIV-1 Vif forms an E3 complex to promote the degradation of A3G. Schematic representation of the E3 ubiquitin ligase complex formed by HIV-1 Vif, with motifs reported to be required for the interaction with Cul5 and EloBC indicated. Adapted from reference [18].

The HIV-1 encoded Vif protein is required for virus infectivity and serves as a specificity factor to the EloBC-Cul5-Rbx2 E3 core. Its substrates are the cellular intrinsic restriction factors APOBEC3G (A3G) and APOBEC3F (A3F), which are targeted by this E3 ligase for poly-ubiquitination and degradation in the proteasome [8], [9], [10] (Figure 1). Several motifs in the N-terminal portion of Vif have been shown to be necessary for its interaction with A3G and/or A3F [11], [12], [13], [14], [15] (summarized in Figure S1), but this interaction remains poorly understood at the molecular level. Vif binds to EloC via its BC-Box [9] (Figure 1 and S1), and interacts with Cul5 via a unique zinc-finger motif upstream of the BC-box [16], [17], [18], [19], [20]. HIV-1 Vif also contains a well-conserved Pro-Pro-Leu-Pro sequence (PPLP) downstream of the BC-Box (Figure 1 and S1), that is essential for Vif function [21], but its precise role remains unclear. Specifically, contradictory reports have linked this motif to the multimerization of Vif [22], or to the interaction with Cul5 [20], A3G [23] or HIV-1 reverse transcriptase [24]. This PPLP motif is followed by a stretch of 12 conserved residues (Figure S1), but these can be removed without impairment to Vif function [21].

Studying the biochemical properties of the SOCS-box of Vif could provide clues as to how this protein contributes to the formation of a functional E3 ligase, as well as shed light on how cellular factors can form similar E3 complexes. From this perspective, we applied a range of structural, biochemical and genetic methods to characterize the interaction between Vif and EloBC. We were able to show that the BC-box of Vif is unstructured in the unbound state, and forms an α-helix only upon binding to EloC, as previously indicated [25]. We also demonstrated a novel interaction between the PPLP motif of Vif and the EloB subunit of EloBC. This interaction requires the binding of the BC-box of Vif to EloC, and is coupled to the folding of an α-helix at the C-terminus of EloB. Finally, we also confirmed that the integrity of the PPLP motif is essential for the assembly of biologically active complexes containing Vif and Cul5 [20]. Altogether, these data indicate that the Vif specificity factor binds to the EloBC complex by an induced-folding mechanism. In addition, they reveal a previously unknown interaction between EloB and Vif, which may also be important for the binding of other specificity factors.

## Results

### Biochemical characterization of the Vif-EloBC interaction

**Figure 2:**
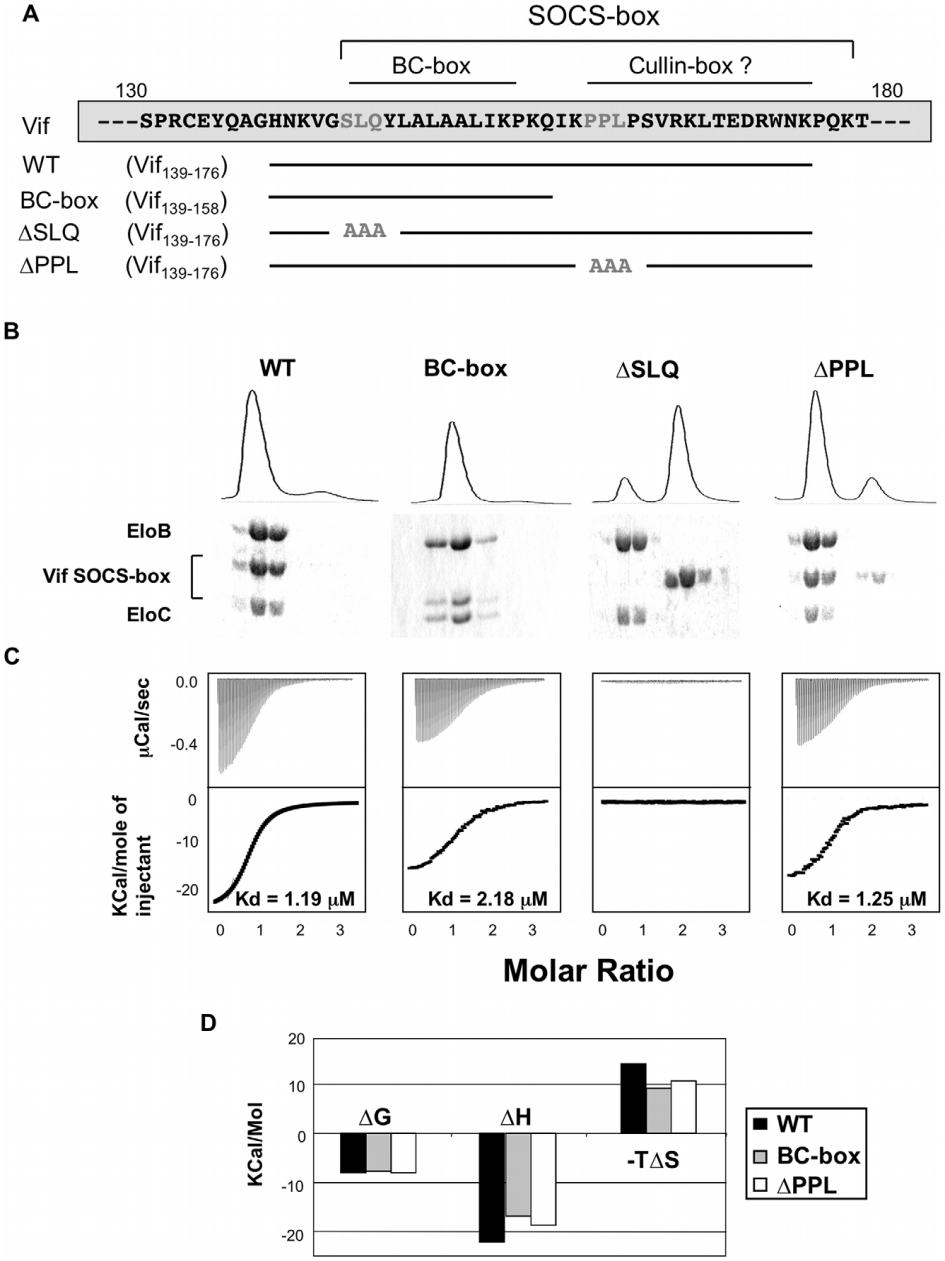
Biochemical analysis of the Vif-EloBC interaction. (A) Schematic representation of the Vif SOCS-box constructs and mutants used in this study. The amino acid sequence is indicated on top, with the mutated residues in gray. (B) Gel filtration binding assay. The Vif SOCS-box and mutated variants were mixed with equimolar amounts of EloBC and run through a gel filtration column, with the UV_280_ trace shown on top. The eluted fractions were collected and run on a 16% polyacrylamide gel and stained with Coomassie blue (bottom). (C) ITC binding assay. The Vif fusion proteins were titrated against EloBC. The raw data are shown on top, the heat integration at the bottom. The resulting K_d_ is given for each construct, except for the ΔSLQ protein, for which no binding was observed. (D) Thermodynamic analysis of the ITC binding assay. The binding free energy (ΔG), enthalpy (ΔH) and entropy (ΔS) are plotted for the Vif fusions proteins binding to EloBC. The ΔSLQ protein is not shown as no binding was observed.

The lack of solubility of the HIV-1 Vif protein has impaired its biochemical and structural characterization [26]. In order to overcome this issue, we expressed and purified an extended region of Vif containing the SOCS-box (residues 139–176) fused to a solubility enhancement tag (SET) [27] at the N-terminus, and a hexahistidine tag at the C-terminus (subsequently referred to as the wild type Vif SOCS-box protein) (Figure S2A). In addition, we engineered several mutated derivatives of this fusion, as summarized in Figure 2A: two known biologically inactive mutations, corresponding to triple alanine substitutions in the BC-box (ΔSLQ) or the PPLP motif (ΔPPL), and a C-terminal truncation to the boundary of the BC-box (referred to as the BC-box protein). As expected, all these proteins eluted as monomers in gel filtration (data not shown), and the monomeric state of the wild type protein was further confirmed by analytical ultracentrifugation (Figure S2B). From these results, we conclude that the PPLP motif is not sufficient to induce multimerization of Vif, contrary to a previous report [22].

We then used a gel filtration assay to monitor binding of the Vif SOCS-box protein to EloBC purified from bacteria [7] (Figure S2A). As shown in Figure 2B, the wild type protein co-eluted with EloBC, at the elution volume expected for a 1∶1∶1 Vif∶EloB∶EloC complex, and SDS-PAGE of the elution fractions, as well as analytical ultracentrifugation of the complex (figure S2D), confirmed this stoichiometry. As expected, the ΔSLQ protein eluted later than EloBC (Figure 2B), indicating that this protein is unable to bind to and form a stable complex with EloBC, confirming the essential role of the BC-box motif in binding to EloBC. The ΔPPL protein, as well as the BC-box protein, also co-eluted with EloBC (Figure 2B), confirming that the BC-box of Vif is sufficient for EloBC binding in this assay.

We subsequently used isothermal titration calorimetry (ITC) to quantify the interaction between Vif and EloBC. Titration of the wild type protein against EloBC could be fitted to a single binding model, with a dissociation constant (K_d_) of 1.2 µM (Figure 2C), which is in the lower range of typical values for protein-protein interactions [28]. As expected, the ΔSLQ protein did not show any significant binding (Figure 2C). The BC-box protein had slightly lower affinity (K_d_ = 2.2 µM), but the ΔPPL protein displayed an affinity similar to the wild type (1.25 µM). This suggests that the modest but reproducible affinity decrease measured for the BC-box protein may not be a specific effect of binding, but rather may reflect size-related properties of the truncation, possibly related to the diffusion rate in solution [29].

Importantly, the changes of enthalpy and entropy upon binding were lower for the BC-box protein than for the wild type protein (Figure 2D). Significantly, the ΔPPL protein also showed decreased changes in enthalpy and entropy compared to wild type, indicating that the PPLP motif contributes to EloBC binding but does not affect the overall dissociation constant for this interaction.

### The BC-box of Vif is unstructured in the absence of EloBC

**Figure 3:**
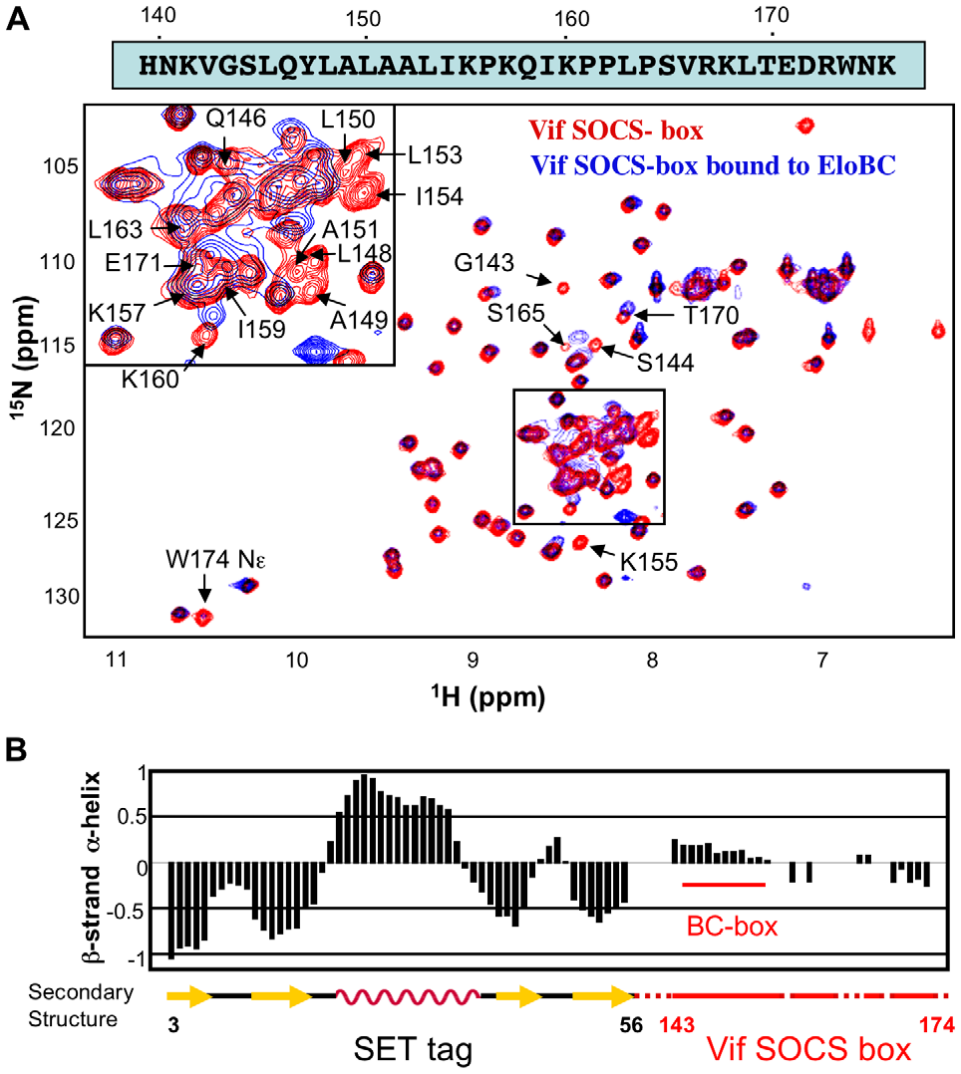
NMR spectroscopy of the Vif SOCS-box. (A) ^15^N-HSQC spectrum of Vif SOCS-box protein in the free state (red spectrum) and bound to EloBC (blue spectrum), with the sequence of the Vif SOCS-box shown on top. The peaks assigned to residues belonging to the Vif SOCS-box are indicated; some peaks are omitted for clarity. (B) Secondary structure of the Vif SOCS-box wild type protein, measured with the software SSP using chemical shifts values. The portion of this protein corresponding to the Vif SOCS-box is indicated in red, with the location of the BC-box indicated. The numbering indicated below corresponds to the SET tag (black) and the Vif sequence (red).

A recently published crystal structure of the Vif BC-box in complex with EloBC [25] confirmed that the BC-box assumes an α-helical conformation when bound to EloBC, and contacts EloC via hydrophobic interactions. We therefore used NMR spectroscopy to determine the conformation of the SOCS-box of Vif when unbound, and to investigate binding to EloBC at the amino acid level. The ^15^N-HSQC spectrum of the wild type Vif SOCS-box protein is shown in Figure 3A (red spectrum); this spectrum is consistent with a well-folded protein, having a good dispersion of peaks between 6 ppm and 11 ppm in the ^1^H dimension. However, comparison of these data to a spectrum of the SET tag alone [30] revealed that most peaks are formed by residues belonging to the tag and not to Vif. A range of triple-resonance and residue-specific labelling experiments allowed us to assign the resonances corresponding to the backbone atoms of approximately 85% of the Vif portion of the fusion protein (Figure 3A), which was confirmed by comparing ^15^N-HSQC spectra of the mutant proteins described above (data not shown). Interestingly, all the peaks assigned to the Vif SOCS-box are located between 7.9 and 8.4 ppm in the ^1^H dimension. Spectra of this nature are characteristic of a random coil conformation, where residues are not held in defined structures [31].The lack of secondary structure of the Vif SOCS-box was then confirmed by measuring the chemical shift deviation, using the software SSP [32], as shown in Figure 3B.

### Structural changes in Vif's SOCS-box when bound to EloBC

We next recorded a ^15^N-HSQC spectrum for a complex of the ^15^N-labelled wild type Vif SOCS-box protein with unlabelled EloBC (spectrum shown in blue in Figure 3A). While most of the peaks corresponding to the SET tag are identical to the unbound spectrum, the peaks corresponding to the BC-box are now absent (Table S1). As the BC-box of Vif is known to be helical when complexed with EloBC [25], we anticipated that new peaks corresponding to the helical conformation of BC-box residues might appear. The absence of peaks, even in excess of EloBC, indicates that the Vif-EloBC complex is dynamic, with conformational fluctuation in the millisecond-to-microsecond range. As a consequence, the residues involved in the interaction could not be observed in ^15^N-HSQC spectra. We note, however, that the dynamic property of this interaction may be caused by the SET-tag and may not hold true for the full-length, untagged Vif protein.

**Figure 4:**
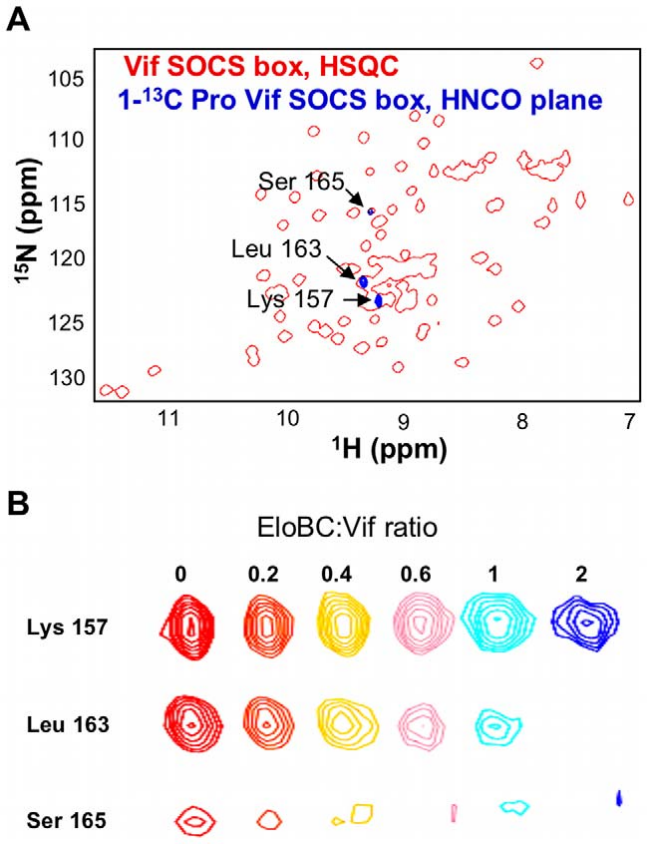
Proline labelling of the Vif SOCS-box protein. (A) NH plane of the HNCO spectrum collected on ^15^N, 1-^13^C proline-labelled Vif SOCS-box protein (blue spectrum), overlapped with the ^15^N-HSQC spectrum of this protein (red spectrum). (B) Titration of EloBC against proline-labelled Vif SOCS-box, with the ratio of EloBC to Vif shown on top. The peak for Leu 163 disappears upon binding, but the peak for Lys 157 is not affected. The peak for Ser 165 also decreases in intensity, and is shifted upfield on both proton and nitrogen dimensions.

It is interesting to note that the peak assigned to Leu-163, the leucine of the PPLP motif, also appears to be missing in the bound spectrum (Figure 3A). As this peak is located in a highly overlapped region of the spectrum, we used residue-specific labelling to study this residue. In particular, the Vif SOCS-box protein was uniformly ^15^N-labelled in the presence of 1-^13^C proline. This results in the carboxyl group of proline residues being ^13^C-labelled, meaning that only residues immediately C-terminal to prolines are observed in HNCO spectra [33] (Figure 4A). NMR spectra confirmed unambiguously that the intensity of the peak corresponding to Leu-163 (and to a lesser extent Ser-165) strongly decreased upon EloBC binding (Figure 4B), indicative of their participation in the interaction. In contrast, the peak corresponding to Lys-157, located between the BC-box and the PPLP motif, was not affected upon binding. This confirms the involvement of the PPLP motif of Vif in binding to EloBC, as suggested by the ITC experiments described above.

### EloC becomes structured upon binding to the BC-box of Vif

**Figure 5:**
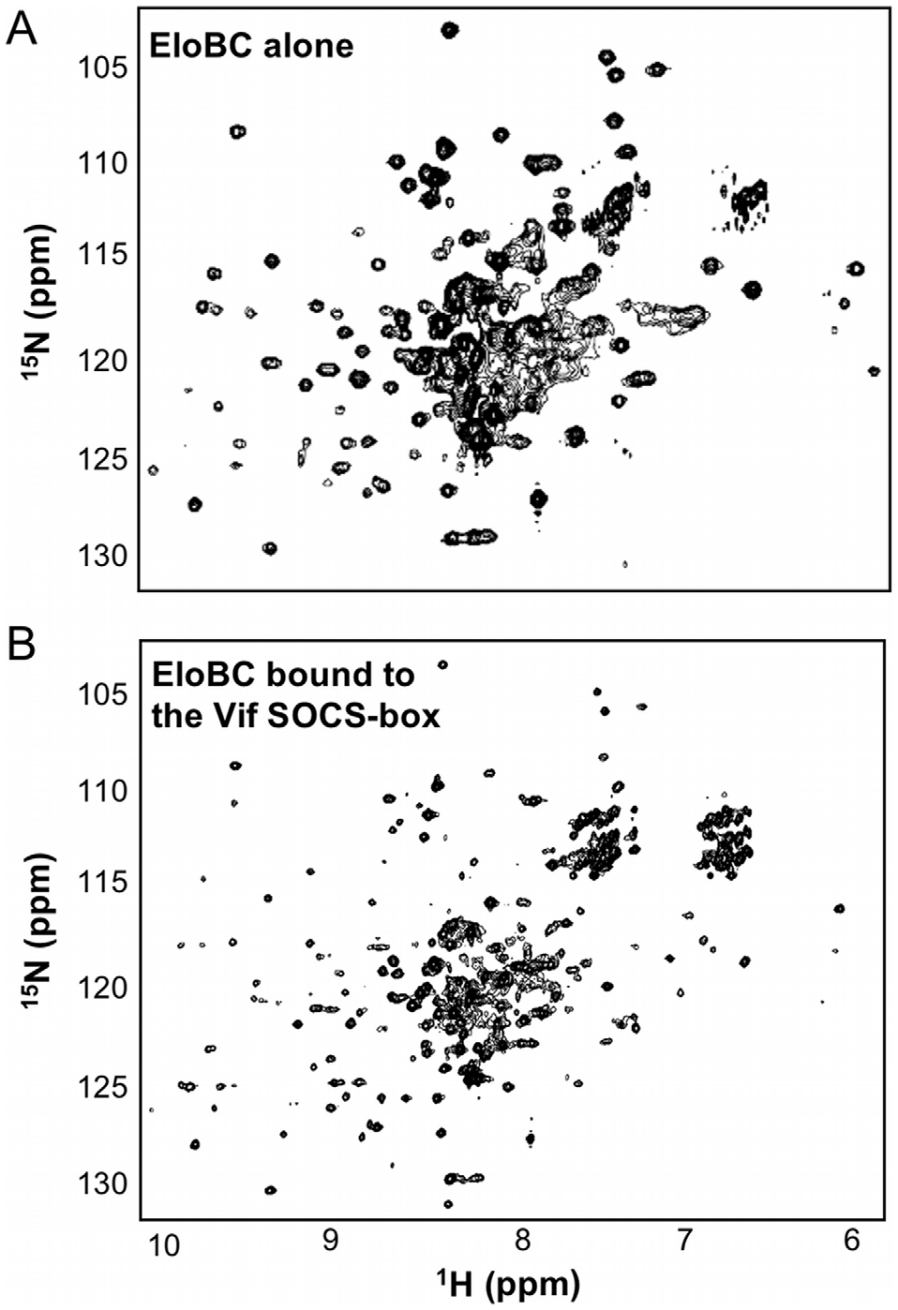
NMR spectroscopy of EloBC. ^15^N-HSQC spectrum of protonated EloBC (A) or uniformly-deuterated EloBC bound to unlabelled wild type Vif SOCS-box protein (B). The best spectra available were used for illustration, but similar spectra were recorded on several occasions using different protein preparations. The spectrum of free EloBC shows some well dispersed peaks, but also significant overlap exists between 7.5 and 8.5 ppm in the ^1^H dimension, indicative of an unstructured domain. The spectrum of bound EloBC is much better resolved, with more peaks corresponding to well-folded domains.

We then investigated the structural changes that occur in EloBC upon binding to Vif. The ^15^N-HSQC spectrum of unbound EloBC indicates that the complex is only partly folded, with some unresolved resonances at the centre of the spectrum (Figure 5A). Analytical ultracentrifugation of EloBC demonstrated that this complex forms a heterodimer in solution (Figure S2C), confirming that the unresolved resonances are not caused by aggregation. It should be mentioned, however, that these data were best fitted to a model in which some residual, non-interacting aggregate impurities are present (Figure S2C). Recent NMR studies of the EloBC complex showed a similar ^15^N-HSQC spectrum for unbound EloBC, and revealed that most of the well-resolved peaks correspond to the ubiquitin-like domain of EloB, while the majority of the peaks for EloC were missing [7].

The spectrum of EloBC bound to the wild type Vif SOCS-box protein is characteristic of a well-folded protein complex (Figure 5B), and is similar to the spectrum of EloBC bound to the BC-box of SOCS3 [7]. This therefore indicates that EloC acquires much of its structure upon ligand binding. The spectrum of EloBC bound to the ΔPPL mutant is similar to the spectrum bound to wild type (Figure S3), demonstrating that the structural reorganization of EloC upon binding is driven by the interaction between EloC and the BC-box of Vif.

### The PPLP motif of Vif interacts with the C-terminus of EloB

One prominent aspect of the ^15^N-HSQC spectrum of unbound EloBC, which has not been reported previously, is the presence of some very intense and poorly dispersed peaks, indicative of residues that are in random-coil conformation and are highly dynamic in the pico- to -nanosecond timescale. Triple-resonance experiments allowed us to assign these peaks to the C-terminal tail of EloB, specifically residues 101 to 118 (Figure S4A). Differences in the buffer and/or temperature used for these experiments likely explain why these peaks were not observed by others [7]. It is worth noting that in the crystal structure of EloBC in complex with SOCS2 [5], as illustrated in Figure S4B, there is no electron density for residues 106 to 118 of EloB, consistent with the presence of significant disorder in this region, but residues 101 to 104 form a well-structured α-helix. The spectrum reported here suggests that these four residues are unstructured; we therefore propose that this α-helix is not formed in unliganded EloBC.

In the ^15^N-HSQC spectrum of EloBC bound to Vif, the intensity of the peaks assigned to the C-terminus of EloB was significantly decreased, particularly for residues 101 to 104 (Figure 5A, 5C and 5D). An NMR titration of unlabelled wild type Vif SOCS-box protein against ^15^N-labelled EloBC (Figure 5A) confirmed that this decrease in intensity occurs upon binding. For residues Asp-101, Val-102, Met-103 and Lys-104, the changes in peak intensity upon titration of the Vif SOCS-box protein could be fitted to K_d_ values of 1.71 µM, 0.43 µM, 1.73 µM and 3.56 µM respectively (Figure 5B), which are close to the K_d_ value obtained by ITC of 1.20 µM. Taken altogether, these data demonstrate that the decrease in intensity of the peaks corresponding to residues 101 to 104 of EloB is caused by binding to Vif.

**Figure 6:**
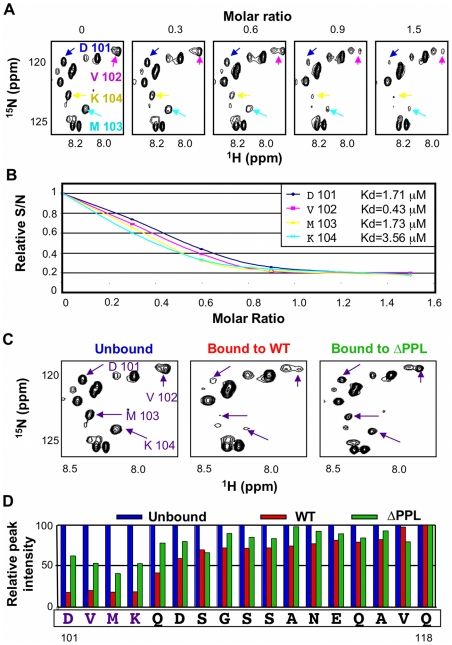
The PPLP motif of Vif interacts with residues 101–104 of EloB. (A) Unlabelled Vif SOCS box protein was titrated against ^15^N-labelled EloBC, and ^15^N-HSQC spectra were recorded for each point of the titration. For these spectra, the contrast is set in order to visualize only the very intense peaks assigned to the C-terminus of EloB. (B) The relative intensities of the peaks assigned to residues 101–104 of EloB were fitted to corresponding K_d_ values using a script written with the Mathematica software. The K_d_ values thus obtained are comparable to the values obtained by ITC. (C) ^15^N-HSQC NMR spectrum of EloBC alone (left panel), bound to the wild type Vif SOCS-box protein (middle panel) or to the ΔPPL protein (right panel); these spectra are shown with a low contrast in order to visualize only the very intense peaks. (D) For each peak assigned to the C-terminus of EloB, the S/N was measured using SPARKY and plotted as a relative intensity compared to the S/N of peaks in the unbound protein.

A decrease in peak intensity in ^15^N-HSQC spectra could be explained by: (1) line broadening upon binding, owing to the increase in complex size and/or changes in dynamic properties of the residue forming this peak, or (2) by the appearance of a new peak representing changed conformation in the bound state. In order to discriminate between these two possibilities we measured relaxation times for peaks assigned to residues 101 to 118 of EloB, and the resulting T_1_/T_2_ ratios are shown in Figure S5: the peak intensities and fitting parameters are given in Table S2. This ratio has been shown to be an accurate measurement of the dynamic properties of individual amino acids [34]. It is evident that this T_1_/T_2_ ratio is higher in the EloBC-Vif complex for residues 106 to 111, indicating that the decrease in peak intensity upon binding observed for these residues is due to line broadening, presumably caused by the increase in molecular weight. In contrast, for residues 101 to 103 the T_1_/T_2_ ratios are identical for the free and bound states of EloBC (Figure S5), indicating that there is no significant broadening of the peaks upon binding for residues 101 to 103, and therefore that altered conformation underlies the decreases in their intensity. From this observation, it can be concluded that the peaks observed for these three residues in the spectrum of EloBC bound to the Vif SOCS-box protein correspond to a fraction of EloB where these residues do not adopt their bound conformation. Since the Vif SOCS-box-EloBC complex was purified in excess of Vif, this observation is unlikely to be caused by the presence of free EloBC, and most likely reveals the transient nature of the PPLP-mediated interaction. Specifically, we propose that residues 101–104 of EloB form an α-helix upon binding to Vif, reminiscent of that found in the crystal structures of EloBC bound to SOCS proteins [5], [6]. In the spectrum of EloBC bound to the ΔPPL protein, the intensity of these four peaks is also reduced, but to a significantly lesser extent, particularly for residues 101 to 104 (Figure 6C right panel, and Figure 6D), and we attribute this to peak broadening rather than new peak formation. These data suggest that residues 101 to 104 of EloB interact, most likely directly, with the SOCS-box of Vif, and that binding is dependent on the integrity of the PPLP motif.

**Figure 7:**
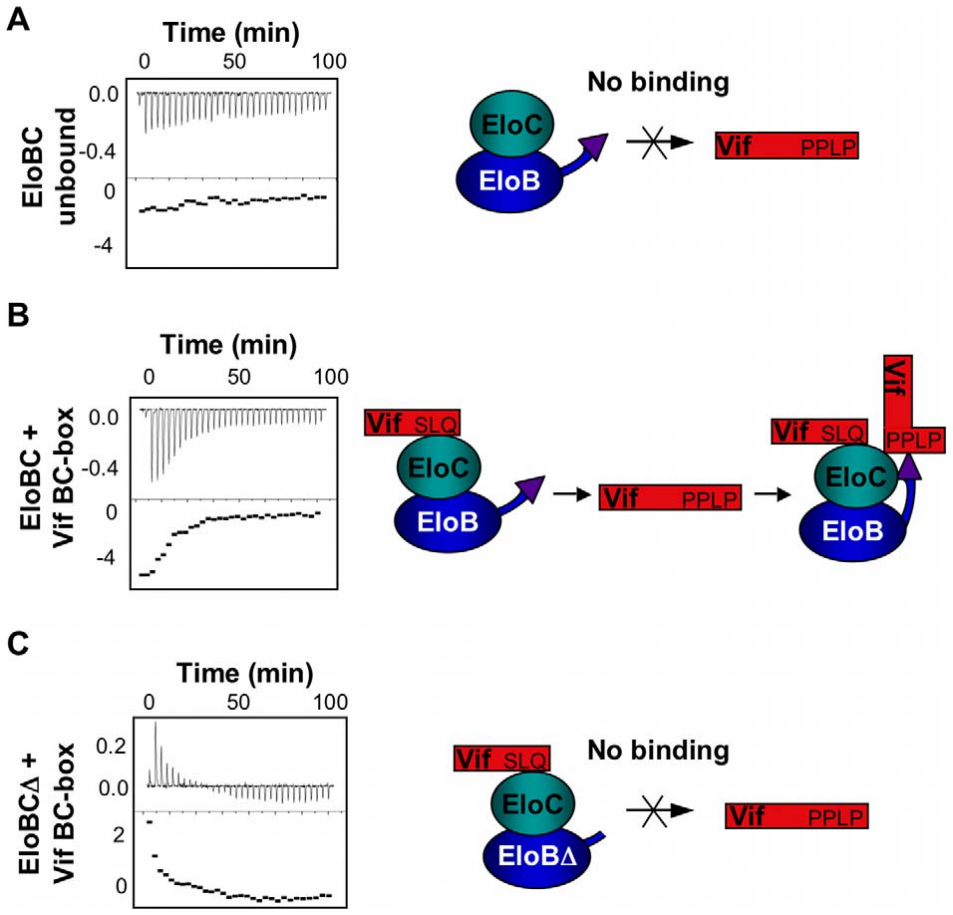
ITC measurement of the Vif-EloB interaction. For all panels, ITC data is shown on the left, with the raw data on top and the heat integration at the bottom. A schematic representation of the experiment is shown on the right. (A) Titration of EloBC complex against the ΔSLQ protein; no binding is observed. (B) Titration of the EloBC/Vif BC-box complex against the ΔSLQ protein. (C) Titration of the EloBCΔ/Vif BC-box complex against the ΔSLQ protein; no binding is measured, but an endothermic reaction is observed instead.

As demonstrated previously, EloBC does not show any significant binding to the SOCS-box of Vif when the SLQ motif is mutated to AAA. We therefore postulated that the interaction between EloB and the PPLP motif of Vif is dependent upon the binding of EloBC to the BC-box of Vif (as illustrated on Figure 7). To test this, we hypothesized that EloBC bound to the BC-box of Vif should be able to bind to the PPLP motif when provided separately. Using ITC, we were able to measure the binding of a complex between EloBC and the BC-box protein to a BC-box deficient Vif fusion protein (i.e., the ΔSLQ protein with an intact PPLP motif) (Figure 7B); neither EloBC alone (Figure 7A) nor the BC-box protein alone (Figure S6A) gave any measurable binding to the BC-box deficient protein. Accordingly, this experiment demonstrated that the PPLP motif of Vif forms a second interaction with EloBC, but only when EloBC had already engaged the BC-box, The specificity of this interaction was further demonstrated by performing this experiment using a double-mutant Vif SOCS-box protein in which the SLQ and PPLP motifs were both disrupted (Figure S6B). As anticipated, no interaction with the preformed EloBC-Vif BC-box complex occurred, confirming the requirement for an intact PPLP motif.

To confirm that this interaction is driven by residues 101 to 104 of EloB, we engineered a deletion of the C-terminal 17 residues of EloB (EloB_1–101_); this deletion did not compromise either the formation or the solubility of the EloBC complex (hereafter referred to as EloBCΔ) (Figure S6C). Similar to full length EloBC, EloBCΔ bound to the wild type Vif SOCS-box protein but not to the ΔSLQ protein (data not shown, Figure S6D). However, the complex of EloBCΔ bound to the BC-box protein failed to show additional binding to the ΔSLQ protein, as measured by ITC (Figure 7C). We note that some energy release was observed for the first few measurements, possibly caused by destabilization of the EloBCΔ-BC-box complex (Figure 7C). In summary, these data confirm that the interaction between the PPLP motif of Vif and EloBC is dependent on residues 101 to 104 of EloB (summarized in Figure 7, right panels).

### The interaction between the PPLP motif and EloB is necessary for the formation of a functional E3 ubiquitin ligase complex

**Figure 8:**
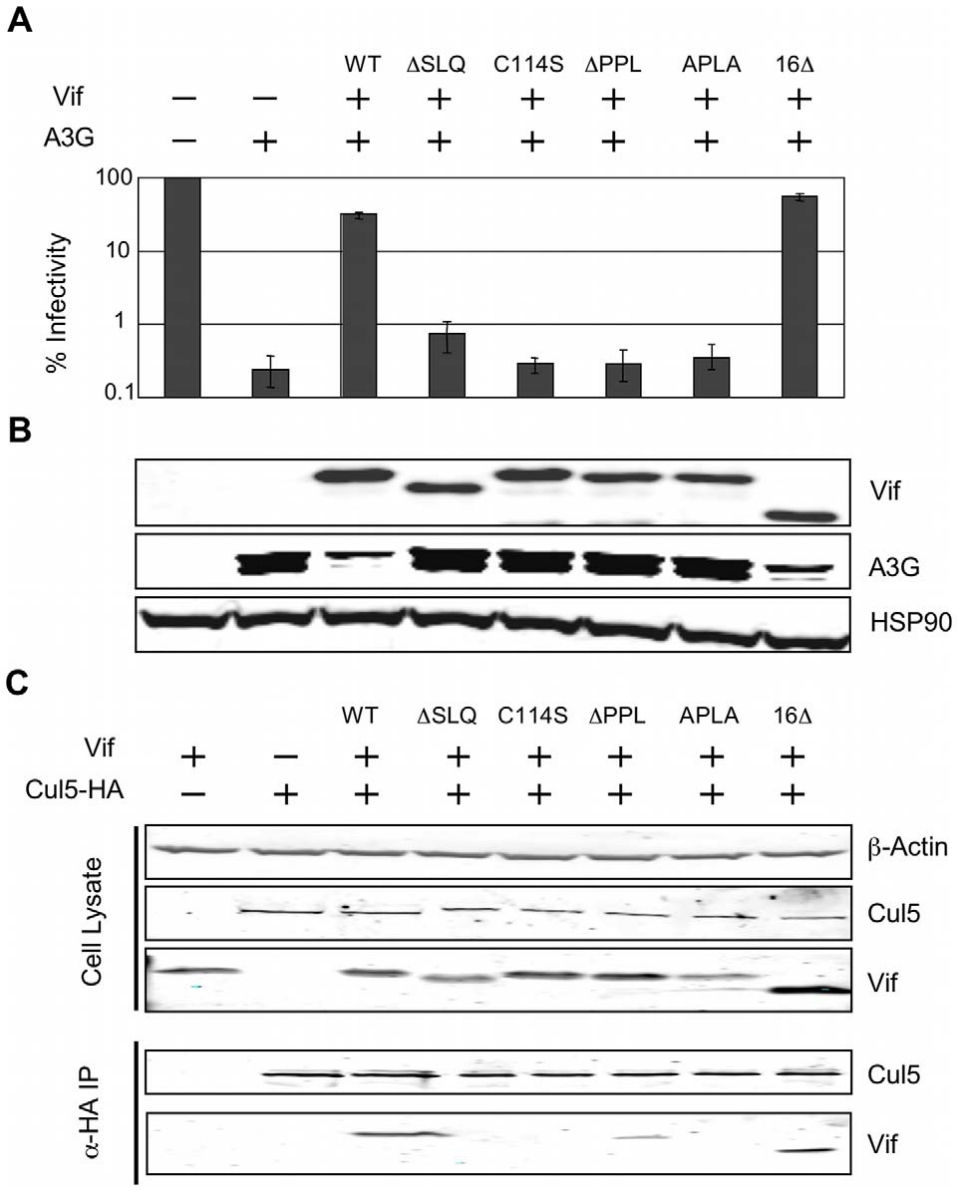
The interaction between the PPLP motif of Vif and EloB is necessary for binding to Cul5. (A) The activity of Vif proteins was measured by the capacity to overcome the anti-viral action of A3G in a single-cycle infectivity assay. The data are represented as relative β-galactosidase units normalised to HIV-1 with no A3G or Vif. The means of three experiments was used, with error bars representing the standard deviation. (B) Immunoblot of the whole cell lysates from the producer cells used for infectivity assays. HSP90 is shown as a loading control. (C) Co-immunioprecipitation of Vif with Cul5. 293T cells were transfected with plasmids expressing HA-tagged Cul5 and wild type or mutated Vif proteins, and Cul5-HA was isolated using an α-HA antibody. Cell lysates and immunoprecipitates were analysed by immunoblotting for presence of Vif and Cul5-HA.

We next investigated the relevance of this newly described PPLP-EloB interaction to Vif function in living cells. We tested the functionality of several mutant Vif proteins in single-cycle HIV-1 infectivity assays, where infection requires the degradation of A3G in virus producing cells. As shown in Figure 8A, the ΔPPL mutant of Vif was inactive, as were the previously described ΔSLQ and C114S mutants. A second mutation of this motif, where PPLP was mutated to APLA, also abrogated Vif function, whereas deletion of the residues downstream of the putative cullin-box (Δ16) was inconsequential. As expected, immunoblot analysis of the corresponding cell lysates confirmed that the wild type and Δ16 Vif proteins induced the degradation of A3G, but that all the proteins containing inactivating mutations failed to do so (Figure 7B).

Having demonstrated above that the PPLP motif of Vif forms a secondary site of interaction between Vif and EloB, we postulated that this interaction is required for the recruitment of Cul5. To test this hypothesis, we measured the binding of Vif to HA-tagged Cul5 by co-immunoprecipitation from lysates of transfected cells (in the absence of A3G). As shown in Figure 7C, Cul5 interacts with wild type Vif and the Δ16 truncated protein. Neither the ΔSLQ nor C114S proteins were detected in the recovered fraction, confirming that the recruitment of EloBC via the BC-box, as well as the integrity of the HCCH domain, are both strictly required for binding to Cul5. Neither the ΔPPL nor the APLA protein efficiently co-immunoprecipitated with Cul5, confirming that the PPLP motif is important for the recruitment of Cul5. A faint band could be observed in the recovered fraction for the ΔPPL protein; since we have demonstrated above that this mutation abrogates the interaction between HIV-1 Vif and the C-terminal helix of EloB, it appears that in the context of over-expressed protein minimal binding to Cul5 is still possible for this protein. This would provide an explanation for the discrepancies between the study by Yu *et al.* [20] and the results reported by Donahue *et al.* [23]: the former reported a loss of binding to Cul5 in immunoprecipitation experiments for mutations of the PPLP motif, while the later did not. Given that this weak interaction was not detected with the APLA protein, we suggest that this mutation may also abrogate an interaction between Vif and Cul5. This would be consistent with the model articulated by *Stanley et al.* [25], where the C-terminal proline of the PPLP motif is proposed to contact loop 2 of Cul5.

## Discussion

**Figure 9:**
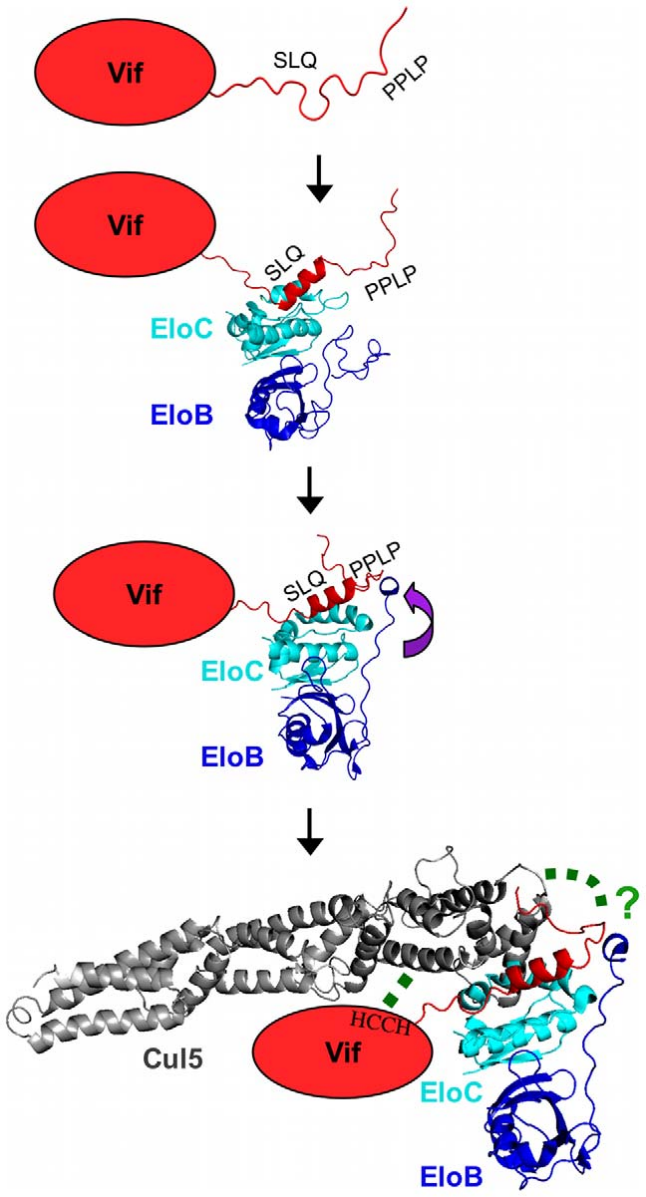
Schematic representation of the induced-folding mechanism for the formation of Vif-EloBC-Cul5 complex. Structure-based representation of the Vif-EloBC-Cul5 complex formation; for simplicity, the various binding events are shown in succession, although in the absence of kinetic data the precise order of events remains to be verified. Vif is represented by a red oval apart from the SOCS-box, which was modelled on the crystal structure of the EloBC-Vif BC-box complex [25] (PDB ID:3DCG). The structure of EloBC bound to the BC-box was modelled on the NMR structure of EloBC bound to the SOCS3 BC-box [7] (PDB ID: 2JZ3). The structure of EloBC bound to the Vif BC-box and the PPLP motif was modelled on the crystal structure of the EloBC-SOCS2 complex [5] (PDB ID:2C9W). The structure of Cul5 was modelled on the crystal structure of Cul1 [46] (PDB ID:1LDJ). The green dashed lines indicate interactions between HIV-1 Vif and Cul5.

Here, we have reported a dissection of the interactions between the HIV-1 Vif protein and the EloBC heterodimer. Our data indicate that at least two binding steps occur, that both induce structural changes in the constituent proteins, and that these changes are necessary for other essential steps in the assembly of a competent E3 ubiquitin ligase complex. This induced-folding mechanism, shown in Figure 9, can be summarized as incorporating the following steps (recognizing that some of these may take place concurrently or consecutively): (1) in the free state, the SOCS-box of HIV-1 Vif is unstructured; (2) when Vif encounters EloBC, its BC-box forms an α-helix and binds to EloC, which also becomes structured (step 1); (3) once Vif is bound to EloC, its PPLP motif interacts with residues 101–104 of EloB, which forms an α-helix (step 2); and (4) this second interaction between Vif and EloBC, together with the interaction between Cul5 and Vif's HCCH domain, is necessary for the recruitment of Cul5 and the assembly of a functional ubiquitin ligase.

These studies were made possible by our capacity to isolate soluble Vif peptides and EloBC independently, and then to reconstitute binding interactions *in vitro*. In this regard, these studies are fundamentally distinct from previous analyses of interactions between EloBC and SOCS proteins [7] or the VHL protein [35] where complex formation necessitated co-expression of the components, thus precluding experiments of the genre discussed here. It is not known if this requirement of co-expression for complex formation observed *in vitro* correlates to a functional property of these proteins *in vivo*. However, it is worth noting that the VHL protein, a specificity factor for an E3 core comprising EloBC, cullin 2 and Rbx1, has been reported to require the chaperone TRiC in order to bind to EloBC *in vivo* [36], providing a potential explanation for the lack of binding reported for proteins purified separately. It is tempting to speculate that the capacity of HIV-1 Vif to bind to EloBC *in vitro* reflects a functional property of this protein such as independence from co-factor/chaperone involvement: this may be indicative of a mechanism by which Vif is able to out-compete other specificity factors to form an E3 ligase complex.

Both NMR and ITC demonstrated that the PPLP motif of Vif interacts specifically with residues 101–104 of EloB. The canonical SOCS-box motif possesses a similar LPLP motif [3], and one question that arises is whether this motif also interacts with EloB In the crystal structure of the SOCS2-EloBC complex [5], the LPLP motif is located in a hydrophobic pocket formed by EloC and residues 101–104 of EloB (Figure S7), particularly the valine and methionine at positions 102 and 103. Interestingly, it has been shown that the C-terminal tail of EloB up to residue 104 is strictly conserved in all species, including *Drosophila* and *Caenorhabditis* [37], though its role has remained unexplained to date. We therefore propose that the LPLP motif of SOCS-box proteins ultimately binds to EloB in an analogous way to the PPLP motif of HIV-1 Vif, and that these two motifs may play a similar role in recruiting Cul5.

We note that both the *in vitro* binding and the co-immunoprecipitation experiments presented here were performed in the absence of A3G, demonstrating that Vif is capable of recruiting the EloBC-Cul5-Rbx2 complex independently of its interaction with A3G. It is possible, however, that prior binding to A3G may alter the kinetics of the interactions discussed here, but such experiments await the purification of the amino-terminal region of A3G that binds Vif. Similarly, it also remains to be determined whether binding of HIV-1 Vif to A3G can occur in the absence of the E3 ubiquitin ligase complex. Interestingly, it has been reported that the PPLP motif also plays a role in binding to A3G [23], [37], which may suggest that the interaction between Vif and EloBC and/or Cul5 affects binding to A3G.

Altogether, the data presented here suggest a refined model for the formation of the Vif-EloBC-Cul5 E3 ubiquitin ligase complex. We demonstrate that the binding of Vif to EloBC is measurable *in vitro*, unlike other SOCS-box proteins, a feature that could be exploited to develop small molecules that specifically inhibit Vif-EloBC complex formation. In addition, we describe a new interaction between the cullin-box of HIV-1 Vif and the C-terminal tail of EloB, and propose that a similar interaction may occur in all SOCS-box proteins. As such, these observations extend the current understanding of how cullin-RING ubiquitin ligases engage their specificity factors and substrates.

## Materials and Methods

### Plasmids and cloning

Vif SOCS-box fusion proteins were expressed in *E.coli* using pET30-GBFusion1 [27], a vector containing the *GB1* gene at the N-terminus followed by a C-terminal His_6_ tag, separated by a BamHI restriction site. This site was used to insert fragments spanning the HIV-1 (HXB-3 isolate) Vif SOCS-box, or mutated derivatives [21].

EloB and EloC were co-expressed in *E.coli* using the pETDuet-1 vector (Novagen). The sequences coding for full length (residues 1–118) or truncated (1–101) EloB were expressed with an the N-terminal His_6_ tag by insertion between the BamHI and HindIII of MCS1. EloC (residues 16–111) was cloned into the MCS2 using the NdeI and XhoI sites.

For the infectivity assay, the *vif*-deficient HIV-1 provirus, pIIIB/Δ*vif*; the HIV-1 Vif expression vector, pcVIF, its corresponding control, pcΔVIF, as well as the A3G expression vector, pA3G, have been described [38]. Previously defined mutated *vif* genes were introduced into pcVIF [21]. The Cul5-HA expression vector, pCUL5HA, was created by inserting the Cul5 cDNA into the pCMV4HA plasmid.

### Protein expression and purification

The Vif SOCS-box fusion proteins were expressed in *E.coli* strain BL21(DE3), and EloB/EloBΔ and EloC were co-expressed in *E.coli* strain BL21(DE3). Cells were grown in LB with 50 mg/l kanamycin (Vif proteins) or 100 mg/l ampicillin (EloBC) at 37°C until OD_600_≈0.5, and expression was induced by adding 1 mM IPTG for 4 hours. Cells were harvested, resuspended in SOCS buffer (20 mM Tris pH 8.0, 500 mM NaCl, 0.05% sodium azide, protease inhibitor coctail [Roche]) or BC buffer (20 mM Tris pH 8.0, 10 mM imidazole, 0.05% sodium azide, protease inhibitor cocktail [Roche]) for the Vif SOCS-box and EloBC respectively. The suspension was then sonicated, clarified by centrifugation at 45,000 g for 50 min. and the supernatant applied to a Ni-NTA resin column (Novagen) equilibrated with SOCS or BC buffer. The resin was rinsed with SOCS/BC buffer, and then with SOCS/BC buffer supplemented with 50 (or 25) mM imidazole. The protein was eluted using SOCS/BC buffer plus 500 mM imidazole and fractions were analysed by SDS-PAGE, pooled and concentrated using a 3 MWCO centricon (Milipore) up to ≈5mg/ml. The samples were further purified by size-exclusion chromatography, using a Superdex 75 column (GE Healthcare) equilibrated in SOCS/BC buffer.

To form EloBC-Vif SOCS-box complexes, the concentrations of the various purified components were estimated by measuring their OD_280_. They were mixed at a 1∶1∶1 EloB∶EloC∶Vif SOCS-box ratio and run on Superdex 75 gel filtration columns (GE Healthcare) in complex buffer (50 mM Tris pH 7.5, 150 mM NaCl, 0.05% sodium azide, protease inhibitor tablets [Roche]).

For NMR experiments the proteins were expressed in E.coli strain BL21/DE3 in M9 minimal media supplemented with ^15^NH_4_Cl, ^13^C-labelled glucose, ^2^H_2_O, and/or 1-^13^C proline depending on the experiment. The proteins were purified as above.

### Isothermal titration calorimetry

All samples were dialysed into 50 mM Tris pH 7.5, 50 mM NaCl, 0.05% sodium azide supplemented with protease inhibitors [Roche]. ITC was performed using a VP-ITC calorimeter, except for the titration of EloBC-Vif BC-box with the double-mutant SOCS-box, which was performed using a ITC200 calorimeter (both machines are manufactured by MicroCal, Inc., Northampton, MA). For the binding of the Vif SOCS-box protein to EloBC, titrations were performed by injecting 90 aliquots of 6.167 µl of protein at ≈0.2 mM into the chamber containing EloBC at 0.025 mM. For analysing PPLP motif binding, the titrations were performed by injecting 30 aliquots of 9.12 µl of the EloBC-Vif BC-box complex at 0.3 mM into the chamber containing the ΔSLQ Vif SOCS-box protein at 0.02 mM.

### Analytical ultracentrifugation

Sedimentation equilibrium experiments were performed using a Beckman Optima XL-A analytical ultracentrifuge as described previously [39]. Samples were prepared in 50 mM Tris pH 7.5, 50 mM NaCl, 0.05% sodium azide supplemented with protease inhibitors [Roche], and data were acquired as an average of 25 absorbance measurements at a wavelength of 280 nm and a radial spacing of 0.001 cm. Sedimentation equilibrium experiments were performed at 4°C, at concentrations corresponding to A_280_ of 0.4, 0.6 and 0.8, and rotor speeds of 14,000, 17,000 and 20,000 RPM for the Vif SOCS-box and EloBC alone, and 12,000, 14,000 and 16,000 RPM for the Vif SOCS-box-EloBC complex. The buoyant molecular mass M(1-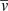
ρ) for a EloBC heterodimer was calculated to be 6965, using a solvent density of 1.00720, 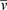 of 0.7233 and a molecular mass of 25656, based on the amino acid composition using SEDNTERP (http://www.rasmb.bbri.org/). Similarly, the buoyant molecular mass M(1-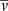
ρ) for a monomer of the Vif SOCS-box was calculated to be 3159, using a solvent density of 1.00720, 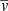 of 0.7263 and a monomer molecular mass of 11767. Data for all three concentrations was analysed simultaneously using a range of models in Sigmaplot, as described previously [39]. Residuals were calculated by subtracting the best fit of the model from the experimental data.

### NMR spectroscopy

For all NMR experiments, protein samples were dialysed in NMR buffer (50 mM phosphate buffer pH 7.0 10% D2O, 0.05 sodium azide, protease inhibitor tablets [Roche]), and concentrated to 0.1–0.5 mM. Approximately 250 µl of samples were transferred to Shigemi tubes for data acquisition.

For the wild type Vif SOCS-box protein, triple-resonance experiments were collected on ^15^N, ^13^C-labelled protein, using a Bruker Avance 700 MHz spectrometer equipped with a cryoprobe. Residue-specific labelling experiments, as well as the spectrum of the Vif SOCS-box in complex with EloBC, were carried out on ^15^N-labelled protein, using a Bruker AvanceIII 600 MHz spectrometer equipped with a cryoprobe.

For EloBC, triple-resonance experiments were collected on ^15^N, ^13^C-labelled protein, using a Bruker Avance 700 MHz spectrometer equipped with a cryoprobe. Spectra of EloBC in complex with the Vif SOCS-box, as well as relaxation data, were acquired on a Varian Inova 800 MHz and a Bruker AvanceII 800 MHz, both equipped with cryoprobes. The ^15^N-HSQC spectra of EloBC bound to the Vif SOCS-box and mutant were acquired on uniformly-deuterated EloBC, using a TROSY-based pulse sequence; relaxation data were acquired on ^15^N-labelled protein.

All spectra were processed with NMRPipe [40] and analyzed with SPARKY [41]. Sequential assignment was carried out using an assignment software promgram ASSIGNER (Laponogov I., unpublished). Relaxation data were fitted using NMRView [42]. NMR titration data were fitted using a script written in the Mathematica software suite.

### Infectivity assay and coimmunoprecipitation

The HIV-1 infectivity assay has been described previously [38]. Briefly, 293T cells were transfected with 1 µg pIIIB/Δ*vif* (provirus), 0.5 µg pA3G and various amounts of pcVIF, or mutant derivatives, to obtain a similar level of expression (the total amount or DNA was balanced to 3 µg with pcΔVif), using polyethylenimine. After 24 h, the supernatant containing HIV-1 was harvested and 50 µl was used to infect 10^5^ TZM-bl indicator cells [43]. The induced expression of β-galactosidase was measured after 24 h using the Galacto-Star system (Applied Biosystems). The producer cells were lysed in SDS-PAGE loading dye for analysis of protein expression by immunoblotting using primary antibodies specific for Vif [44], A3G [45] or HSP90 (Invitrogen). Bound antibodies were detected using the Li-COR technology, with IRDye-labelled secondary antibodies and an Odyssey imager.

For co-immunoprecipitation, 293T cells were transfected with various amount of pcVIF or mutant derivatives, to obtain similar levels of expression (balanced to 3 µg with pcΔVif), and 2 µg pCUL5HA. Cells were lysed in 250 µl IP buffer (50 mM Tris pH 7.0, 150 mM NaCl, 0.5% Triton, protease inhibitor tablets (Roche)), and incubated with Protein G agarose beads (Invitrogen) and rabbit anti-HA antibody (Rockland) at 4°C for 3 h. The beads were washed 4 times with IP buffer and resuspended in 25 µl gel loading buffer. 10 µl of the immunoprecipitated samples, as well as 10 µl of cell lysate, were run on 16% acrylamide SDS-PAGE gel and analysed by immunoblotting using primary antibodies specific for Vif or HA (Covance), and Li-COR technology.

## Supporting Information

Figure S1 HIV-1 Vif sequences alignment. The Vif sequence from representative strains of several HIV-1 clades, along with a Vif sequence derived from an SIV isolated from a chimpanzee, were aligned using ClustalW [47] and the secondary structure was predicted with Phyre [48] (The isolates used are 93BR020, 96CM_MP535, 90CF056, SE9280, ELI, NY5, 92BR025, MAL, 92NG083, CPZ_GAB1, YBF30 and ANT70 for the HIV-1 group M subtypes F1, K, H, J, D, B, C, A, G, the chimpanzee SIV and HIV-1 group N and O Vif sequences, respectively). The figure was generated using ESPript [49]. The N-terminal half (residues 1–99) is predicted to consist primarily of β-sheets, and contains motifs implicated in binding to A3G/A3F. The C-terminal half (residues 100–198) is predicted to be mostly helical and contains the motifs involved in forming the E3 EloBC-Cul5-Rbx2 ubiquitin ligase complex.
(1.02 MB TIF)

Figure S2 Characterization of the Vif SOCS-box protein. (A) SDS-PAGE gel of the purified proteins. For each purified protein used in the binding assay, approximately 10 µg was run on a 16% acrylamide SDS-PAGE gel and stained with Coomassie blue. All proteins appear to be between 95 and 99% pure. (B) Analytical ultracentrifugation of the Vif SOCS-box. The data shown here were collected on a sample with OD_280_ = 0.8, at 20,000, and fitted to a single ideal species with the molecular weight floated. (C) Analytical centrifugation of EloBC. The data shown was collected at loading concentrations corresponding to A_280_ values of 0.8 (circles), 0.6 (squares), and 0.4 (triangles), at 14,000 RPM. A shows the data curves simultaneously fitted to a monomer + aggregate non-interaction model, and gives and excellent fit, with the residuals randomly distributed around zero. The residuals for a single species and a monomer-dimer equilibrium respectively are shown on the right, and it is clear that both of these fits are less good. (D) Analytical centrifugation of EloBC-Vif SOCS-box complex. The data shown was collected at loading concentrations corresponding to A_280_ values of 0.8 (circles), 0.6 (squares), and 0.4 (triangles), at 12,000 RPM. A shows the data curves simultaneously fitted to a monomer + aggregate non-interaction model, and gives and excellent fit, with the residuals randomly distributed around zero. Residuals for a single species and a monomer-dimer equilibrium respectively are shown on the right, and it is clear that both of these fits are less good.
(2.73 MB TIF)

Figure S3 NMR spectrum of EloBC bound to the ΔPPL mutant. ^15^N-HSQC spectrum of uniformly ^15^N, ^2^H-labelled EloBC bound to unlabelled ΔPPL Vif SOCS box protein. This spectrum is similar to the ^15^N-HSQC spectrum of EloBC bound to wild type Vif SOCS box protein (Figure 5B).
(0.38 MB TIF)

Figure S4 The C-terminus of EloB. (A) The ^15^N-HSQC of EloBC (Figure 5A) is shown here at low contrast. Most of the very intense peaks observed were assigned to residues 101 to 118 of EloB. The peaks assigned to residues 101 to 104 are highlighted. (B) The structure of EloBC in complex with SOCS2 is shown (PDB ID: 2C9W), with the α-helix formed by residues 101 to 104 of EloB highlighted. SOCS2 was omitted for clarity.
(0.77 MB TIF)

Figure S5 Relaxation measurements for EloB. T1 and T2 relaxation measurement spectra were recorded on ^15^N-labelled free EloBC and bound to the Vif SOCS-box protein. The T_1_/T_2_ ratios were calculated for the intense peaks corresponding to residues 101–118 of EloB.
(0.26 MB TIF)

Figure S6 Controls for measurements of the Vif-EloB interaction. (A) ITC titration of the Vif BC-box protein against the Vif SOCS-box ΔSLQ protein. No heat release is observed, confirming that the binding recorded with the EloBC-Vif BC-box complex (Fig. 7B) is not caused by the BC-box. (B) ITC titration of an EloBC-Vif BC-box complex against the double mutant (ΔSLQ+ΔPPL) Vif SOCS-box protein. No heat release is observed, confirming that the binding recorded with the Vif SOCS-box ΔSLQ mutant (Fig. 7B) is dependent on the integrity of the PPLP motif. (C) Approximately 10 µg of purified EloBC and EloBCΔ were run on a 16% polyacrylamide SDS-PAGE gel and stained with Coomassie blue. (D) ITC titration of an EloBCΔ-Vif BC-box complex against the Vif SOCS-box ΔSLQ mutant. No heat release is observed, and the endothermic reaction observed with the EloBCΔ-Vif BC-box complex (Fig. 7C) is not present either.
(0.87 MB TIF)

Figure S7 The LPLP motif of SOCS2. The crystal structure of EloBC bound to SOCS2 (PDB ID: 2C9W) is shown, with EloBC in surface representation; hydrophobic areas are shown in red. The side chains of the LPLP motif of SOCS2 are highlighted. It appears that the side chains of this motif are buried in a hydrophobic pocket formed by EloC and the C-terminal helix of EloB, suggesting a binding mode similar to what is proposed for HIV-1 Vif.
(5.38 MB TIF)

Table S1 Chemical shift mapping for the Vif SOCS-box protein upon binding to EloBC. Residues 3 to 58 correspond to the SET tag, and the appended Vif residues, 139 to 171, are numbered according to their position in the full-length Vif sequence and are indicated in bold font. The chemical shifts were measured using Sparky, and the combined chemical shift difference was calculated with the equation **combined shift difference = [(proton shifts)^2^+(nitrogen shifts/6.51)^2^]^0.5^**. Non-assigned residues, as well as residues for which peaks are not observed in the spectrum of the bound protein, are indicated.
(0.11 MB DOC)

Table S2 Relaxation measurements for the C-terminal tail of EloB. The measurement of ^15^N relaxation parameters T_1_ and T_2_, and the T_1_/T_2_ ratio displayed on Figure S5, are given for residues 101–118 of EloB, in the context of free EloBC and EloBC bound to the Vif SOCS-box protein. The fitting of peak intensities was obtained using NMRView.
(0.07 MB DOC)
