# Supplementary figures and images for: The SOCS-Box of HIV-1 Vif Interacts with ElonginBC by Induced-Folding to Recruit Its Cul5-Containing Ubiquitin Ligase Complex
Source: PLoS Pathog. 2010 Jun 3;6(6):e1000925. doi: 10.1371/journal.ppat.1000925 (PMC2880568; doi:10.1371/journal.ppat.1000925)

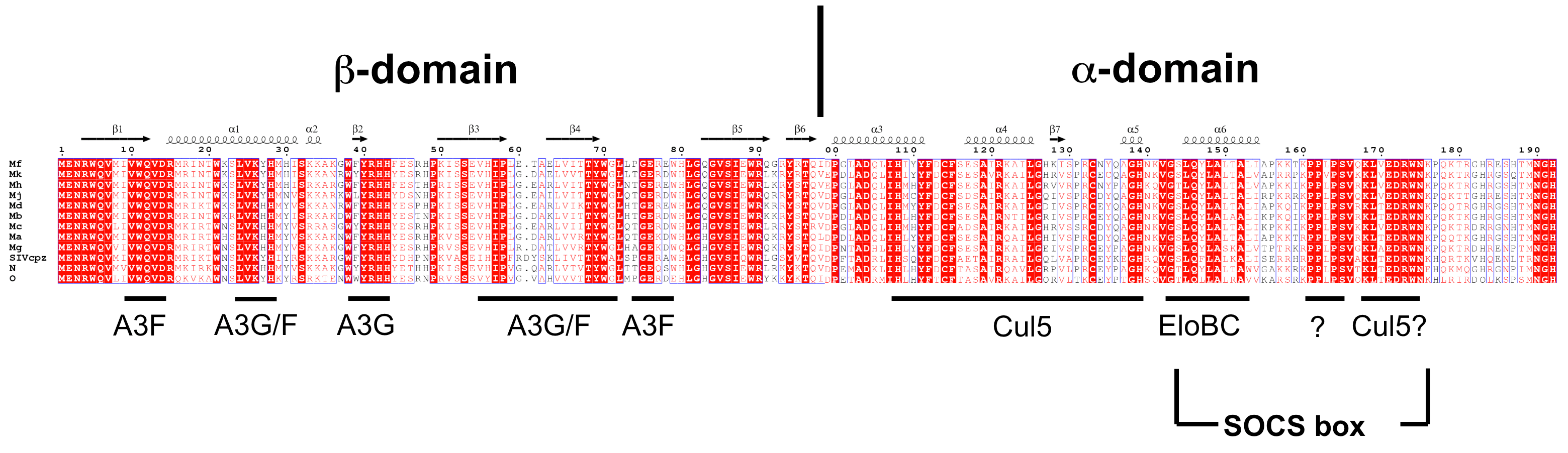

Supplement: Figure S1 — HIV-1 Vif sequences alignment. The Vif sequence from representative strains of several HIV-1 clades, along with a Vif sequence derived from an SIV isolated from a chimpanzee, were aligned using ClustalW [47] and the secondary structure was predicted with Phyre [48] (The isolates used are 93BR020, 96CM_MP535, 90CF056, SE9280, ELI, NY5, 92BR025, MAL, 92NG083, CPZ_GAB1, YBF30 and ANT70 for the HIV-1 group M subtypes F1, K, H, J, D, B, C, A, G, the chimpanzee SIV and HIV-1 group N and O Vif sequences, respectively). The figure was generated using ESPript [49]. The N-terminal half (residues 1–99) is predicted to consist primarily of β-sheets, and contains motifs implicated in binding to A3G/A3F. The C-terminal half (residues 100–198) is predicted to be mostly helical and contains the motifs involved in forming the E3 EloBC-Cul5-Rbx2 ubiquitin ligase complex. (1.02 MB TIF) [file ppat.1000925.s001.tif]

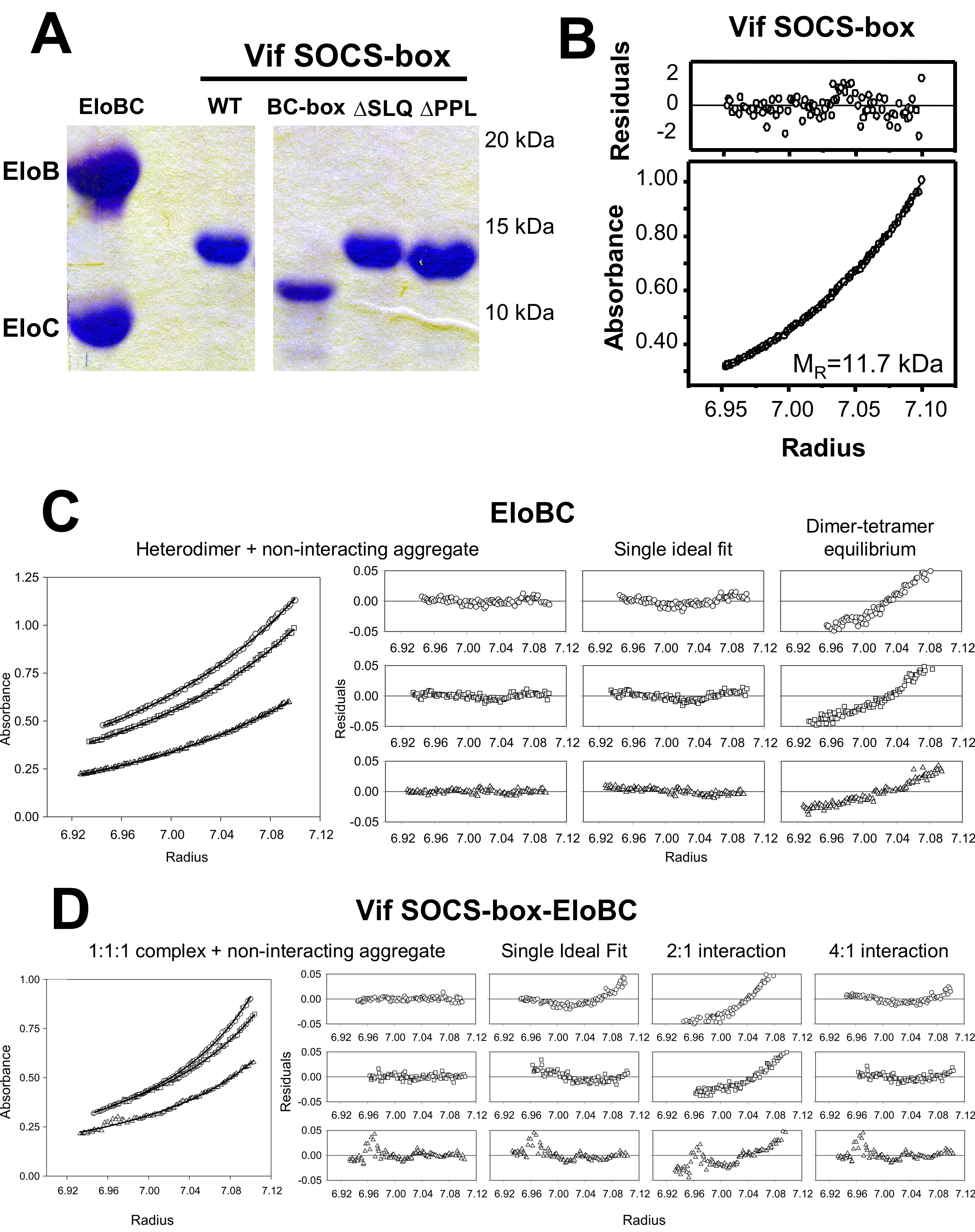

Supplement: Figure S2 — Characterization of the Vif SOCS-box protein. (A) SDS-PAGE gel of the purified proteins. For each purified protein used in the binding assay, approximately 10 µg was run on a 16% acrylamide SDS-PAGE gel and stained with Coomassie blue. All proteins appear to be between 95 and 99% pure. (B) Analytical ultracentrifugation of the Vif SOCS-box. The data shown here were collected on a sample with OD280 = 0.8, at 20,000, and fitted to a single ideal species with the molecular weight floated. (C) Analytical centrifugation of EloBC. The data shown was collected at loading concentrations corresponding to A280 values of 0.8 (circles), 0.6 (squares), and 0.4 (triangles), at 14,000 RPM. A shows the data curves simultaneously fitted to a monomer + aggregate non-interaction model, and gives and excellent fit, with the residuals randomly distributed around zero. The residuals for a single species and a monomer-dimer equilibrium respectively are shown on the right, and it is clear that both of these fits are less good. (D) Analytical centrifugation of EloBC-Vif SOCS-box complex. The data shown was collected at loading concentrations corresponding to A280 values of 0.8 (circles), 0.6 (squares), and 0.4 (triangles), at 12,000 RPM. A shows the data curves simultaneously fitted to a monomer + aggregate non-interaction model, and gives and excellent fit, with the residuals randomly distributed around zero. Residuals for a single species and a monomer-dimer equilibrium respectively are shown on the right, and it is clear that both of these fits are less good. (2.73 MB TIF) [file ppat.1000925.s002.tif]

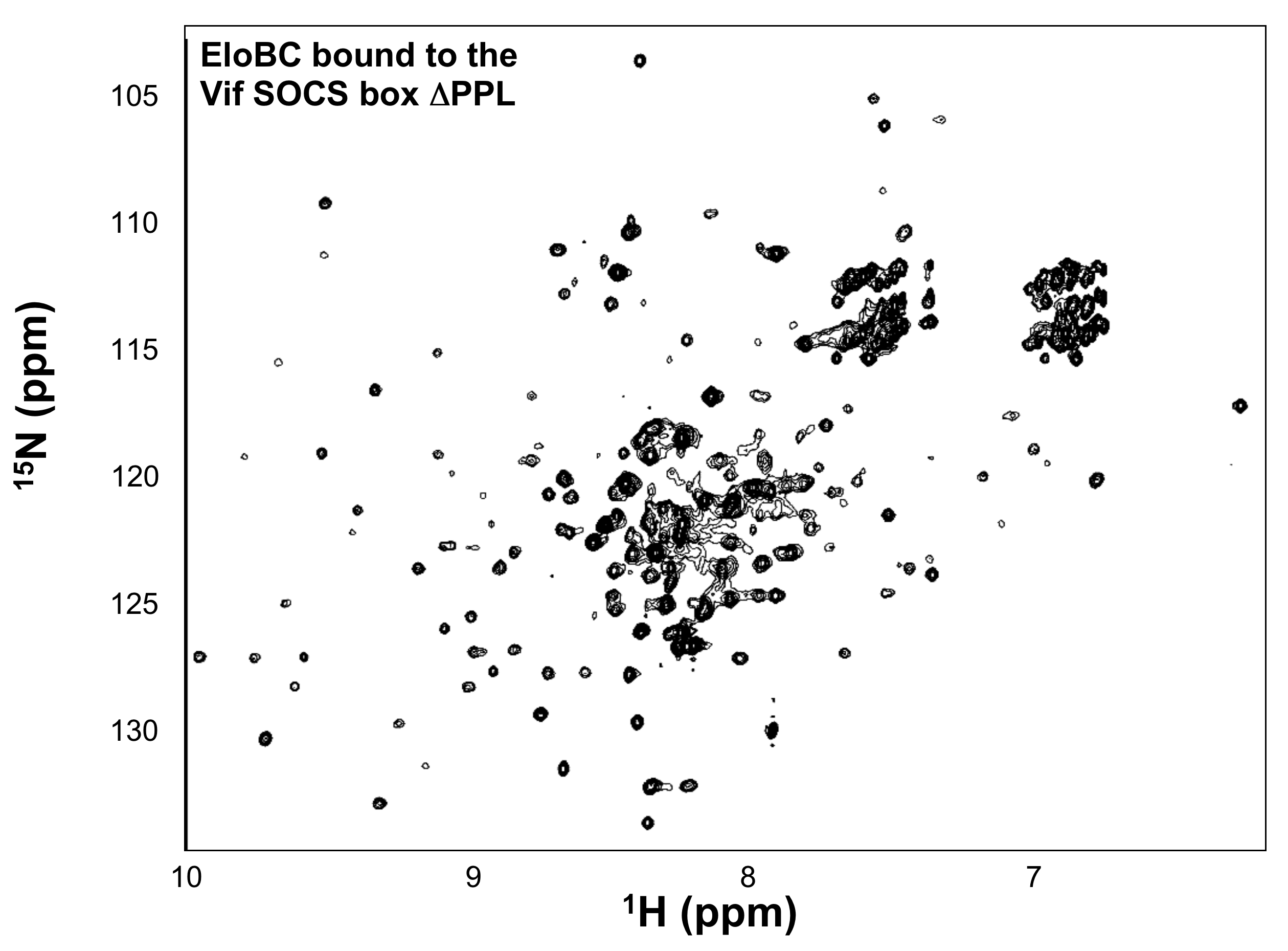

Supplement: Figure S3 — NMR spectrum of EloBC bound to the ΔPPL mutant. 15N-HSQC spectrum of uniformly 15N, 2H-labelled EloBC bound to unlabelled ΔPPL Vif SOCS box protein. This spectrum is similar to the 15N-HSQC spectrum of EloBC bound to wild type Vif SOCS box protein (Figure 5B). (0.38 MB TIF) [file ppat.1000925.s003.tif]

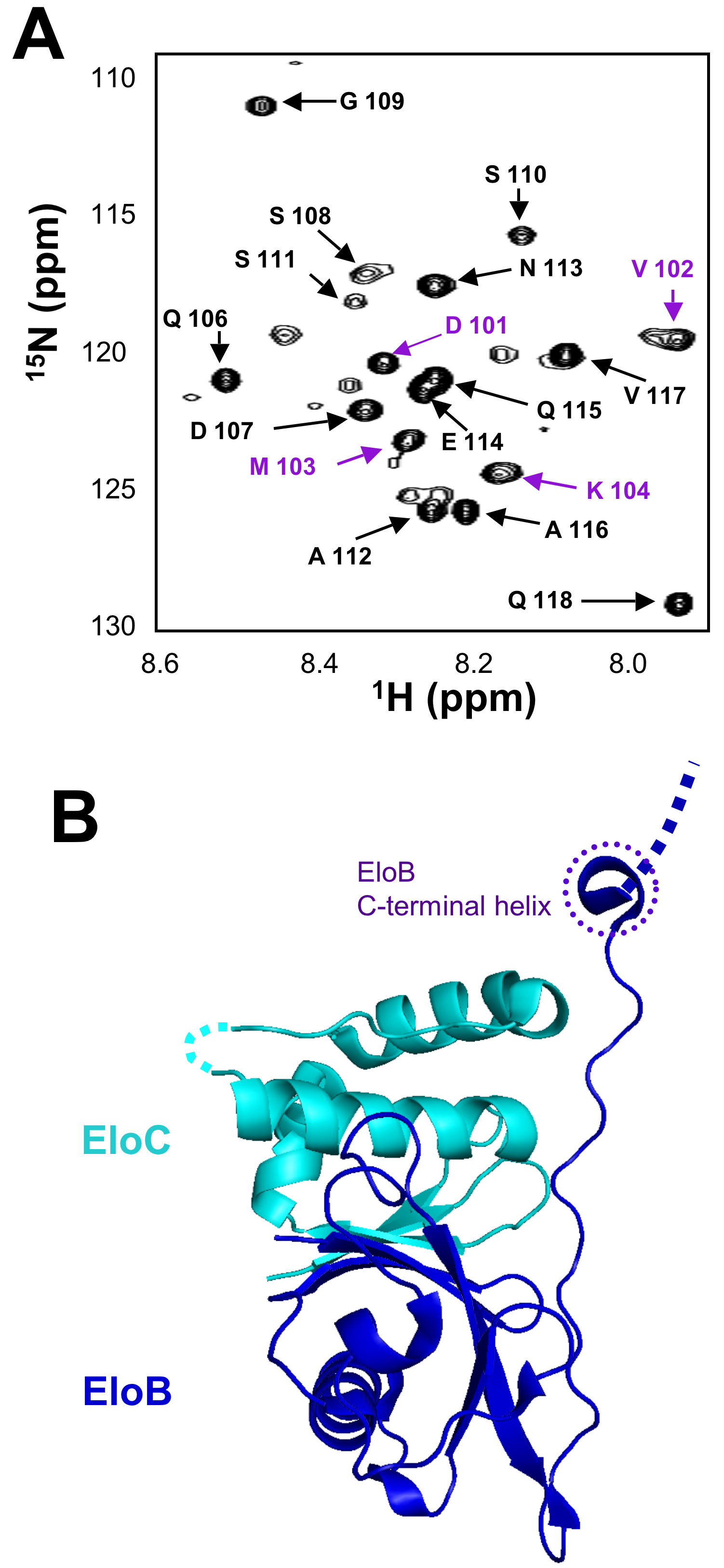

Supplement: Figure S4 — The C-terminus of EloB. (A) The 15N-HSQC of EloBC (Figure 5A) is shown here at low contrast. Most of the very intense peaks observed were assigned to residues 101 to 118 of EloB. The peaks assigned to residues 101 to 104 are highlighted. (B) The structure of EloBC in complex with SOCS2 is shown (PDB ID: 2C9W), with the α-helix formed by residues 101 to 104 of EloB highlighted. SOCS2 was omitted for clarity. (0.77 MB TIF) [file ppat.1000925.s004.tif]

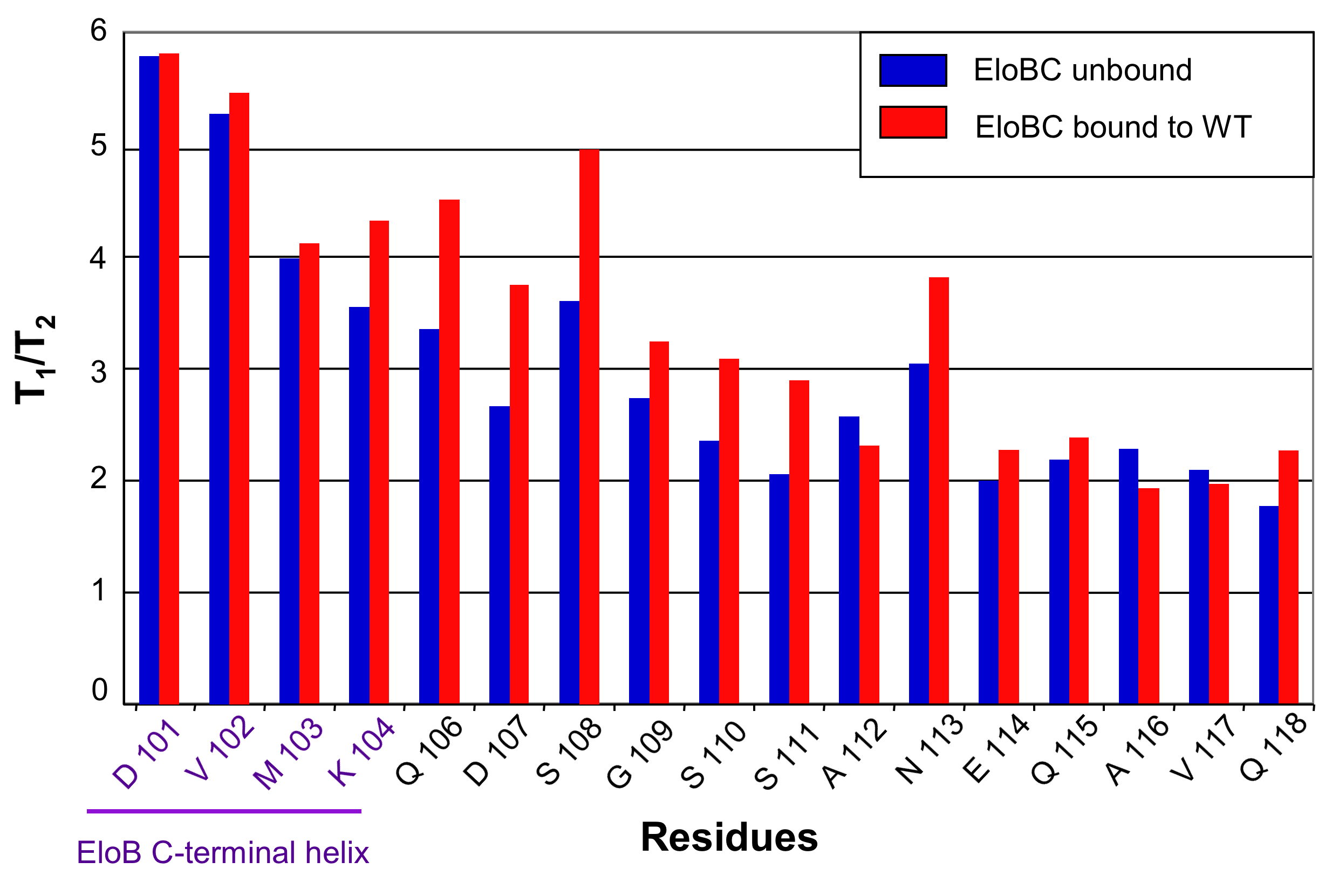

Supplement: Figure S5 — Relaxation measurements for EloB. T1 and T2 relaxation measurement spectra were recorded on 15N-labelled free EloBC and bound to the Vif SOCS-box protein. The T1/T2 ratios were calculated for the intense peaks corresponding to residues 101–118 of EloB. (0.26 MB TIF) [file ppat.1000925.s005.tif]

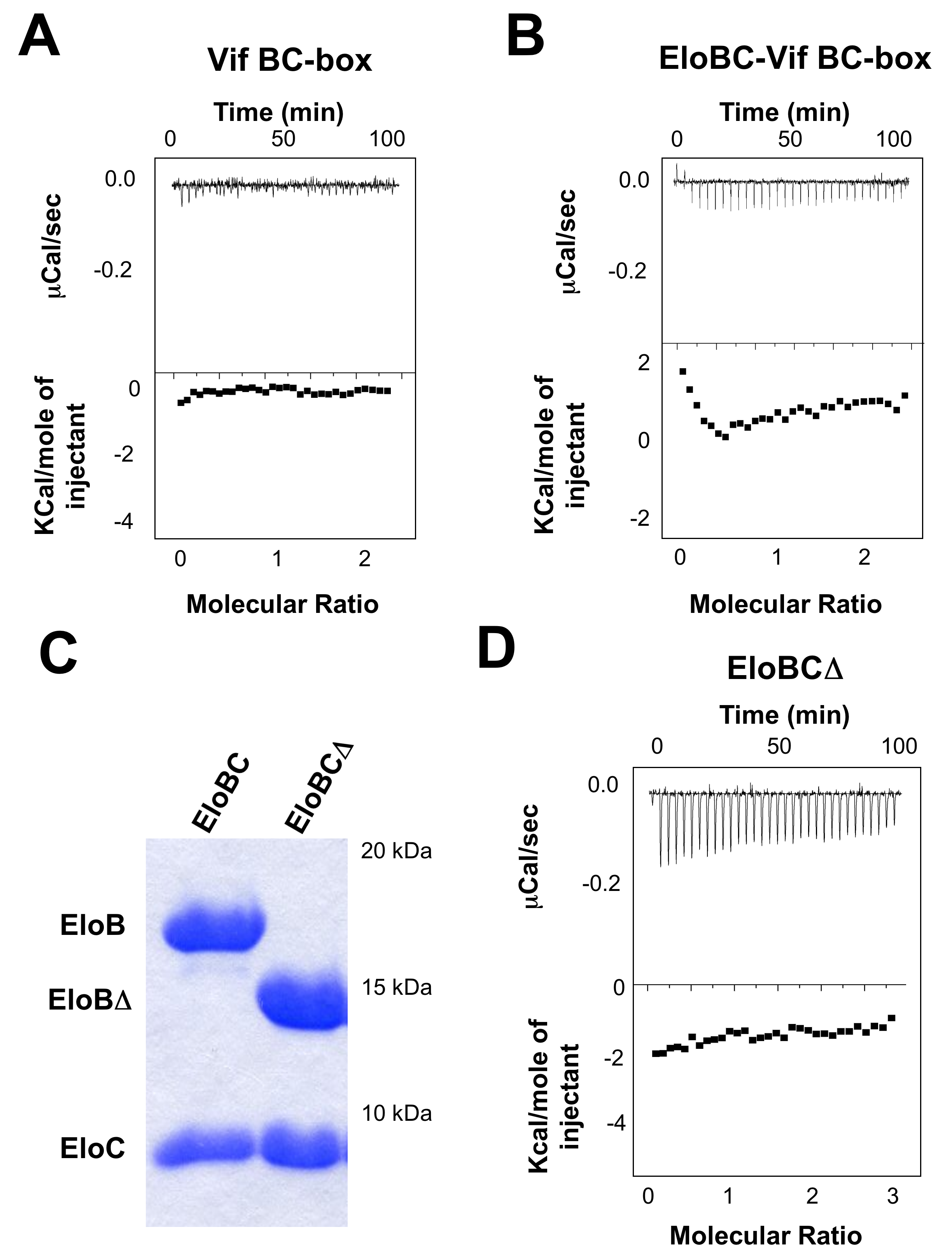

Supplement: Figure S6 — Controls for measurements of the Vif-EloB interaction. (A) ITC titration of the Vif BC-box protein against the Vif SOCS-box ΔSLQ protein. No heat release is observed, confirming that the binding recorded with the EloBC-Vif BC-box complex (Fig. 7B) is not caused by the BC-box. (B) ITC titration of an EloBC-Vif BC-box complex against the double mutant (ΔSLQ+ΔPPL) Vif SOCS-box protein. No heat release is observed, confirming that the binding recorded with the Vif SOCS-box ΔSLQ mutant (Fig. 7B) is dependent on the integrity of the PPLP motif. (C) Approximately 10 µg of purified EloBC and EloBCΔ were run on a 16% polyacrylamide SDS-PAGE gel and stained with Coomassie blue. (D) ITC titration of an EloBCΔ-Vif BC-box complex against the Vif SOCS-box ΔSLQ mutant. No heat release is observed, and the endothermic reaction observed with the EloBCΔ-Vif BC-box complex (Fig. 7C) is not present either. (0.87 MB TIF) [file ppat.1000925.s006.tif]

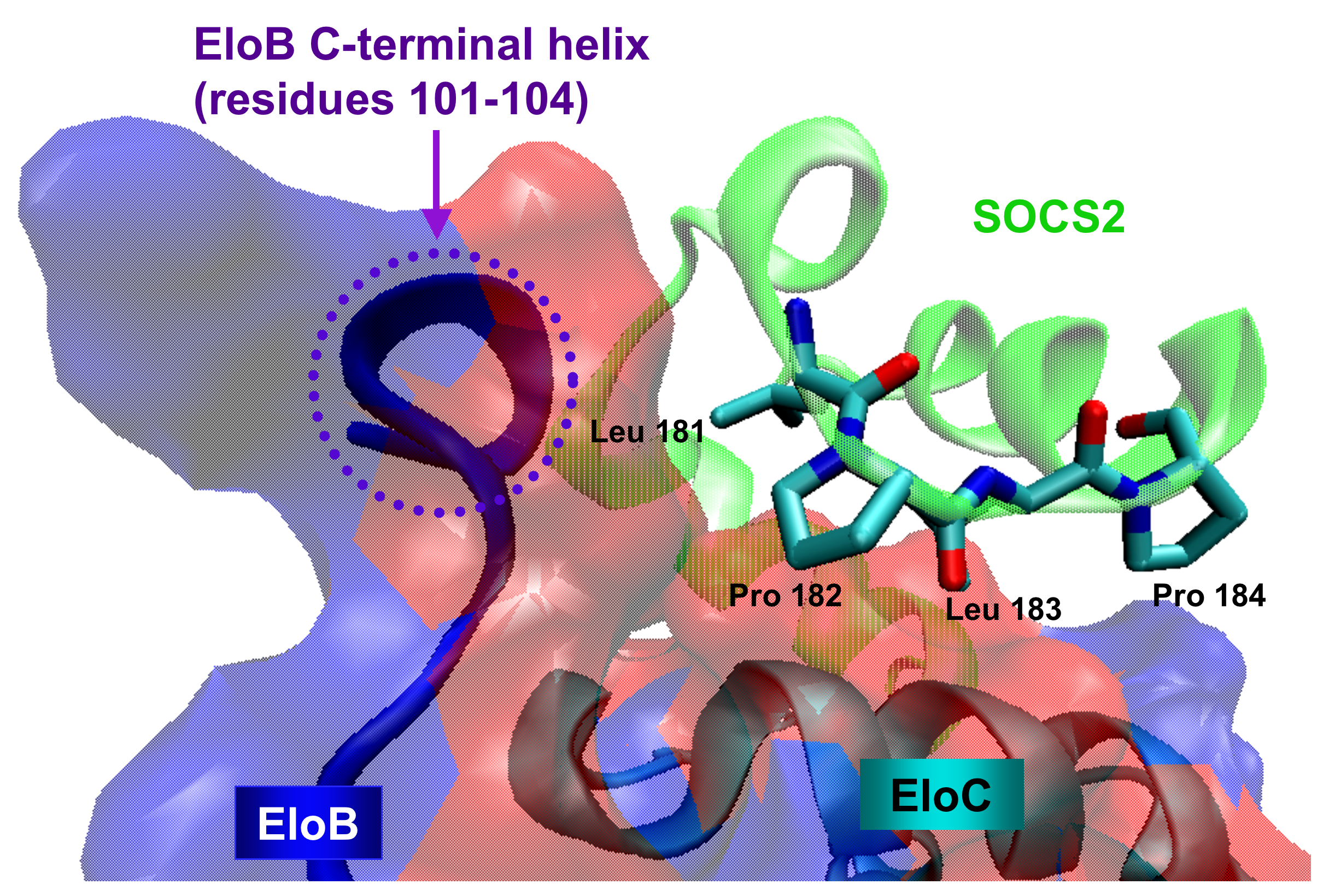

Supplement: Figure S7 — The LPLP motif of SOCS2. The crystal structure of EloBC bound to SOCS2 (PDB ID: 2C9W) is shown, with EloBC in surface representation; hydrophobic areas are shown in red. The side chains of the LPLP motif of SOCS2 are highlighted. It appears that the side chains of this motif are buried in a hydrophobic pocket formed by EloC and the C-terminal helix of EloB, suggesting a binding mode similar to what is proposed for HIV-1 Vif. (5.38 MB TIF) [file ppat.1000925.s007.tif]
