# Supplementary material for: The SOCS-Box of HIV-1 Vif Interacts with ElonginBC by Induced-Folding to Recruit Its Cul5-Containing Ubiquitin Ligase Complex
Source: PLoS Pathog. 2010 Jun 3;6(6):e1000925. doi: 10.1371/journal.ppat.1000925 (PMC2880568; doi:10.1371/journal.ppat.1000925)
Supplement: Table S1 — Chemical shift mapping for the Vif SOCS-box protein upon binding to EloBC. Residues 3 to 58 correspond to the SET tag, and the appended Vif residues, 139 to 171, are numbered according to their position in the full-length Vif sequence and are indicated in bold font. The chemical shifts were measured using Sparky, and the combined chemical shift difference was calculated with the equation combined shift difference = [(proton shifts)2+(nitrogen shifts/6.51)2]0.5. Non-assigned residues, as well as residues for which peaks are not observed in the spectrum of the bound protein, are indicated. (0.11 MB DOC) [file ppat.1000925.s008.doc]

| **Residue** | **Chemical shift difference** |
| --- | --- |
|  |  |
| Tyr 3 | 0.037 |
| Lys 4 | 0.041 |
| Leu 5 | 0.040 |
| Ile 6 | 0.040 |
| Leu 7 | 0.060 |
| Asn 8 | 0.019 |
| Gly 9 | 0.042 |
| Lys 10 | 0.031 |
| Thr 11 | 0.053 |
| Leu 12 | 0.050 |
| Lys 13 | 0.039 |
| Gly 14 | 0.042 |
| Glu 15 | 0.024 |
| Thr 16 | 0.057 |
| Thr 17 | 0.048 |
| Thr 18 | 0.017 |
| Glu 19 | 0.050 |
| Ala 20 | 0.038 |
| Val 21 | 0.045 |
| Asp 22 | 0.032 |
| Ala 23 | 0.046 |
| Ala 24 | 0.059 |
| Thr 25 | 0.071 |
| Ala 26 | 0.042 |
| Glu 27 | 0.065 |
| Lys 28 | 0.040 |
| Val 29 | 0.040 |
| Phe 30 | 0.023 |
| Lys 31 | 0.025 |
| Gln 32 | 0.042 |
| Tyr 33 | 0.026 |
| Ala 34 | 0.041 |
| Asn 35 | 0.045 |
| Asp 36 | 0.041 |
| Asn 37 | 0.076 |
| Gly 38 | 0.033 |
| Val 39 | 0.057 |
| Asp 40 | 0.035 |
| Gly 41 | 0.050 |
| Glu 42 | 0.045 |
| Trp 43 | 0.051 |
| Thr 44 | 0.002 |
| Tyr 45 | 0.019 |
| Asp 46 | 0.033 |
| Asp 47 | 0.041 |
| Ala 48 | 0.040 |
| Thr 49 | 0.004 |
| Lys 50 | 0.040 |
| Thr 51 | 0.037 |
| Phe 52 | 0.040 |
| Thr 53 | 0.029 |
| Val 54 | 0.025 |
| Thr 55 | 0.035 |
| Glu 56 | 0.036 |
| Gly 57 | 0.063 |
| Ser 58 | Not assigned |
| **His 139** | Not assigned |
| **Asn 140** | Not assigned |
| **Lys 141** | Not assigned |
| **Val 142** | Not assigned |
| **Gly 143** | Missing in bound spectrum |
| **Ser 144** | Missing in bound spectrum |
| **Leu 145** | Missing in bound spectrum |
| **Gln 146** | Missing in bound spectrum |
| **Tyr 147** | Missing in bound spectrum |
| **Leu 148** | Missing in bound spectrum |
| **Ala 149** | Missing in bound spectrum |
| **Leu 150** | Missing in bound spectrum |
| **Ala 151** | Missing in bound spectrum |
| **Ala 152** | Missing in bound spectrum |
| **Leu 153** | Missing in bound spectrum |
| **Ile 154** | Missing in bound spectrum |
| **Lys 155** | 0.127 |
| **Pro 156** | Not assigned |
| **Lys 157** | 0.073 |
| **Gln 158** | Not assigned |
| **Ile 159** | 0.081 |
| **Lys 160** | 0.108 |
| **Pro 161** | Not assigned |
| **Pro 162** | Not assigned |
| **Leu 163** | Missing in bound spectrum |
| **Pro 164** | Not assigned |
| **Ser 165** | 0.153 |
| **Val 166** | 0.074 |
| **Arg 167** | Not assigned |
| **Lys 168** | Not assigned |
| **Leu 169** | 0.064 |
| **Thr 170** | 0.095 |
| **Glu 171** | 0.035 |
