# Supplementary material for: The SOCS-Box of HIV-1 Vif Interacts with ElonginBC by Induced-Folding to Recruit Its Cul5-Containing Ubiquitin Ligase Complex
Source: PLoS Pathog. 2010 Jun 3;6(6):e1000925. doi: 10.1371/journal.ppat.1000925 (PMC2880568; doi:10.1371/journal.ppat.1000925)
Supplement: Table S2 — Relaxation measurements for the C-terminal tail of EloB. The measurement of 15N relaxation parameters T1 and T2, and the T1/T2 ratio displayed on Figure S5, are given for residues 101–118 of EloB, in the context of free EloBC and EloBC bound to the Vif SOCS-box protein. The fitting of peak intensities was obtained using NMRView. (0.07 MB DOC) [file ppat.1000925.s009.doc]

|  | Residue | **T1** | | | | **T2** | | | | T1/T2 |
| --- | --- | --- | --- | --- | --- | --- | --- | --- | --- | --- |
|  |  | Value | Max | Min | RMSD | Value | Max | Min | RMSD |  |
|  |  |  |  |  |  |  |  |  |  |  |
| **Free EloBC** | D 101 | 794.519 | 893.824 | 736.747 | 0.072 | 137.222 | 196.927 | 111.798 | 0.179 | 5.790 |
|  | **V 102** | 806.229 | 878.641 | 725.730 | 0.084 | 152.793 | 216.838 | 111.734 | 0.220 | 5.277 |
|  | **M 103** | 674.898 | 763.828 | 591.529 | 0.056 | 169.547 | 326.382 | 125.120 | 0.184 | 3.981 |
|  | **K 104** | 706.965 | 795.107 | 662.307 | 0.079 | 199.304 | 327.971 | 135.990 | 0.242 | 3.547 |
|  | **Q 106** | 737.040 | 794.533 | 658.850 | 0.085 | 219.798 | 452.934 | 138.069 | 0.263 | 3.353 |
|  | **D 107** | 734.021 | 874.349 | 654.584 | 0.097 | 275.789 | 599.914 | 156.672 | 0.272 | 2.662 |
|  | **S 108** | 822.843 | 926.908 | 715.881 | 0.049 | 228.426 | 548.199 | 121.385 | 0.141 | 3.602 |
|  | **G 109** | 767.132 | 879.247 | 667.322 | 0.070 | 280.550 | 141.512 | 167.400 | 0.237 | 2.734 |
|  | **S 110** | 814.800 | 961.059 | 717.599 | 0.063 | 345.284 | 2.661E+07 | 141.435 | 0.207 | 2.360 |
|  | **S 111** | 828.865 | 970.166 | 726.044 | 0.038 | 402.581 | 2.475E+04 | 194.182 | 0.120 | 2.059 |
|  | **A 112** | 775.385 | 868.683 | 658.554 | 0.085 | 301.778 | 862.895 | 178.847 | 0.268 | 2.569 |
|  | **N 113** | 819.827 | 926.026 | 708.584 | 0.124 | 269.214 | 901.555 | 142.417 | 0.373 | 3.045 |
|  | **E 114** | 763.864 | 883.337 | 708.359 | 0.140 | 383.123 | 3.024E+07 | 192.804 | 0.606 | 1.994 |
|  | **Q 115** | 794.198 | 930.708 | 721.710 | 0.190 | 362.923 | 4.382E+06 | 158.780 | 0.678 | 2.188 |
|  | **A 116** | 876.076 | 1.041E+03 | 703.282 | 0.209 | 381.780 | 3.269E+07 | 158.649 | 0.638 | 2.295 |
|  | **V 117** | 1.052E+03 | 1.278E+03 | 895.224 | 0.337 | 499.760 | 3.861E+07 | 210.283 | 1.235 | 2.104 |
|  | **Q 118** | 1.218E+03 | 1.457E+03 | 992.951 | 0.397 | 683.336 | 4.021E+07 | 142.148 | 1.514 | 1.783 |
|  |  |  |  |  |  |  |  |  |  |  |
| **EloBC bound** | **D 101** | 667.390 | 787.567 | 555.501 | 0.014 | 114.760 | 152.014 | 92.416 | 0.017 | 5.816 |
|  | **V 102** | 632.280 | 701.143 | 540.297 | 0.013 | 115.705 | 156.132 | 85.279 | 0.024 | 5.465 |
|  | **M 103** | 500.478 | 597.280 | 398.195 | 0.011 | 121.598 | 142.072 | 96.460 | 0.011 | 4.116 |
|  | **K 104** | 572.838 | 657.921 | 473.408 | 0.012 | 132.572 | 207.825 | 100.489 | 0.020 | 4.321 |
|  | **Q 106** | 679.212 | 776.834 | 593.352 | 0.021 | 150.560 | 253.337 | 111.097 | 0.041 | 4.511 |
|  | **D 107** | 691.487 | 816.329 | 608.259 | 0.020 | 184.362 | 308.887 | 129.180 | 0.035 | 3.751 |
|  | **S 108** | 841.354 | 1.032E+03 | 728.113 | 0.010 | 169.843 | 290.965 | 108.898 | 0.023 | 4.954 |
|  | **G 109** | 719.712 | 828.322 | 610.335 | 0.015 | 221.956 | 443.591 | 148.057 | 0.034 | 3.243 |
|  | **S 110** | 772.096 | 910.895 | 699.523 | 0.009 | 250.047 | 714.483 | 144.564 | 0.027 | 3.088 |
|  | **S 111** | 711.648 | 773.464 | 660.626 | 0.003 | 245.732 | 664.273 | 146.993 | 0.017 | 2.896 |
|  | **A 112** | 714.747 | 822.001 | 611.063 | 0.017 | 308.897 | 3.382E+03 | 162.839 | 0.049 | 2.314 |
|  | **N 113** | 776.733 | 1.015E+03 | 587.666 | 0.028 | 203.790 | 582.320 | 164.201 | 0.048 | 3.811 |
|  | **E 114** | 678.173 | 766.133 | 596.480 | 0.026 | 298.421 | 1.248E+07 | 143.561 | 0.120 | 2.273 |
|  | **Q 115** | 703.492 | 835.957 | 610.576 | 0.040 | 294.140 | 2,375.585 | 152.323 | 0.115 | 2.392 |
|  | **A 116** | 759.925 | 944.949 | 680.121 | 0.035 | 390.183 | 6.885E+07 | 161.263 | 0.112 | 1.948 |
|  | **V 117** | 927.647 | 1.132E+03 | 822.867 | 0.071 | 470.242 | 4.321E+07 | 172.994 | 0.224 | 1.973 |
|  | **Q 118** | 1.086E+03 | 1.323E+03 | 938.575 | 0.069 | 476.413 | 6.074E+07 | 196.651 | 0.222 | 2.280 |
